# Predicting wet age-related macular degeneration (AMD) using DARC (detecting apoptosing retinal cells) AI (artificial intelligence) technology

**DOI:** 10.1080/14737159.2020.1865806

**Published:** 2020-12-28

**Authors:** Paolo Corazza, John Maddison, Paolo Bonetti, Li Guo, Vy Luong, Alan Garfinkel, Saad Younis, Maria Francesca Cordeiro

**Affiliations:** a ICORG, Imperial College London, London, UK; b Western Eye Hospital Imperial College Healthcare NHS Trust, London, UK; c University Eye Clinic, DINOGMI, Polyclinic Hospital San Martino IRCCS, Genoa, Italy; d Novai Ltd, Reading, UK; e UCL Institute of Ophthalmology, London, UK; f Lincoln College, University of Oxford;; g Department of Medicine, UCLA

**Keywords:** DARC, biomarker, AMD, CNV, angiogenesis, SRF

## Abstract

**Objectives:**

To assess a recently described CNN (convolutional neural network) DARC (Detection of Apoptosing Retinal Cells) algorithm in predicting new Subretinal Fluid (SRF) formation in Age-related-Macular-Degeneration (AMD).

**Methods:**

Anonymized DARC, baseline and serial OCT images (n = 427) from 29 AMD eyes of Phase 2 clinical trial (ISRCTN10751859) were assessed with CNN algorithms, enabling the location of each DARC spot on corresponding OCT slices (n = 20,629). Assessment of DARC in a rabbit model of angiogenesis was performed in parallel.

**Results:**

A CNN DARC count >5 at baseline was significantly (p = 0.0156) related to development of new SRF throughout 36 months. Prediction rate of eyes using unique DARC spots overlying new SRF had positive predictive values, sensitivities and specificities >70%, with DARC count significantly (p < 0.005) related to the magnitude of SRF accumulation at all time points. DARC identified earliest stages of angiogenesis in-vivo.

**Conclusions:**

DARC was able to predict new wet-AMD activity. Using only an OCT-CNN definition of new SRF, we demonstrate that DARC can identify early endothelial neovascular activity, as confirmed by rabbit studies. Although larger validation studies are required, this shows the potential of DARC as a biomarker of wet AMD, and potentially saving vision-loss.

## 1. Background

Age-related macular degeneration (AMD) is the leading cause of blindness in developed countries [1] with an incidence that is predicted to rise as the aging population increases [2–5]. Two forms of AMD exist, classified by the presence (‘wet’) or absence (‘dry’) of choroidal neovascularization (CNV) and characterized by the formation of subretinal or intraretinal fluid (SRF or IRF, respectively) from leaking vasculature [6]. Optical coherence tomography (OCT) is currently considered the gold standard for the management of AMD [7] [8], especially when assessing therapy targeting anti-Vascular Endothelial Growth factor (anti-VEGF), an intervention that has revolutionized the treatment of wet AMD [8]. However, early diagnosis and prompt anti-VEGF therapy is a fundamental aspect for the preservation of vision in AMD patients [9].

One approach to preventing vision loss in wet AMD is the identification of patients at risk of developing new CNV [10–13]. As is increasingly utilized in disease prediction, an artificial intelligence (AI) approach was used in these studies, most recently a Convolutional Neural Network (CNN) algorithm [10]. A CNN is a Deep Learning algorithm that can take in an input image, assign importance (learnable weights aclinically should improve disease management and reduce socio-economic burdened biases) to various aspects/objects in the image and be able to differentiate one from the other. AI systems of retinal images are advocated in ophthalmology because they enable reliable and quantitative metrics to be obtained rapidly [14]. Their application clinically should improve disease management and reduce socio-economic burden [15] [16].

We have recently described a retinal imaging technology that combines a CNN-aided algorithm with an intravenously administered novel biologic fluorescent marker, ANX776 (fluorescent-labeled annexin V) which binds to exposed phosphatidylserine on apoptotic and stressed cell membranes [17]. ANX776 positive-labeled cells are visualized as ‘white spots’ on the retina using the ICGA settings of a commonly utilized confocal scanning laser ophthalmoscope (cSLO) in a technology called DARC (Detection of Apoptosing Retinal Cells) [18]. Using the DARC CNN, we reported this technology was able to successfully predict glaucomatous progressive disease as defined by OCT scans with a positive predictive value (PPV) of 100% with all patients with a DARC Count greater than 30 showing significant OCT RNFL (retinal nerve fiber layer) thinning at 18 months.

Based on these very positive results in glaucoma, we have now used the same DARC CNN, with no modifications or retraining, to assess wet AMD disease. Using a cohort of 19 AMD patients from the Phase 2 DARC clinical trial at the Western Eye Hospital, ICHNT, London, we aimed to evaluate the DARC CNN in predicting SRF formation, where SRF was identified by a CNN in serial OCTs up to 36 months after DARC was performed.

## 2. Research design and methods

### 2.1. Study population and setting

A single-center, open-label, Phase 2 clinical trial of DARC was conducted at The Western Eye Hospital, Imperial College Healthcare NHS Trust, London between 15 February 2017 and 30 June 2017. Subjects each received a single intravenous injection of fluorescent annexin 5 (ANX776, 0.4 mg). AMD subjects, already under the care of the medical retina department at the Western Eye Hospital were recruited to the trial, with informed consent being obtained according to the Declaration of Helsinki after the study was approved by the Brent Research Ethics Committee. (ISRCTN10751859). Patients were considered for inclusion in the study if they were eligible according to the inclusion and exclusion criteria outlined in Table 1. Specifically, for inclusion in the AMD cohort, they either had to show CNV (wet AMD) or changes consistent with ‘Early AMD’ (drusen, retinal pigment epithelium (RPE) pigment changes) or ‘Late AMD’ (geographic atrophy of the RPE (dry AMD)). Furthermore, the presence of ocular conditions with increased risk of CNV, and current or past use for more than 30 days of chloroquine, hydroxychloroquine, chlorpromazine, thioridazine, quinine sulfate, clofazimine, cisplatin, carmustine, (BCNU), deferoxamine, amiodarone, isotretinoin, or gold were excluded. Eyes were divided into those with active CNV, previous CNV, early AMD, and late AMD at baseline, based on clinical examination and retinal imaging findings.

**Table 1.** Accuracy of unique DARC spots predicting eyes with new SRF

Time (months)						
Predictive Value % PPV Specificity Sensitivity NPV %	6	12	18	24	30	36
	DARC	DARC	DARC	DARC	DARC	DARC
	71	70	82	77	86	80
	90	86	89	82	86	79
	83	88	82	83	80	80
	95	95	89	88	80	79
Conversion Rate	7.4	11.5	26.1	26.1	29.2	33.3

### 2.2. DARC and OCT image acquisition and study blinding

All participants had DARC performed once only at baseline. Retinal images were acquired with a cSLO (HRA+OCT Spectralis, Heidelberg Engineering GmbH, Heidelberg, Germany), as previously described^8^. Fluorescence settings on the high-resolution ICGA mode were used (diode laser 786 nm excitation; photodetector with 800-nm barrier filter) with baseline infrared autofluorescent images acquired after pupillary dilatation (1% tropicamide and 2.5% phenylephrine). Each patient received ANX776 via intravenous injection (single dose 0.4 mg) and was imaged during and after ANX776 injection at 15, 120, and 240 min, with averaged images from 100 frames recorded at each time point, as previously described [18]. All images were anonymized before any analysis was performed, and only the baseline and 240-min images captured at the level of the RNFL were used in this study.

For analysis of DARC images, the newly described and unmodified DARC CNN algorithm was used to identify DARC spots [17]. Briefly, the DARC CNN algorithm comprised training a CNN to classify spots from a control dataset which was initially identified using template matching and manually classified by five observers. Agreement of 2 out of 5 observers was used to train the CNN, which was found to have an accuracy of 97%, with 91.1% sensitivity and 97.1% specificity^8^. The DARC CNN model and weights that were reported have been used in this work with no modifications. The CNN DARC count was analyzed with respect to the development of eyes with new SRF fluid.

New SRF was identified by OCT analysis. Patients had OCT scans recorded at every follow-up visit, as per standard of care, to a maximum of 36 months after DARC images were obtained. To detect SRF on the OCT, a CNN was used to identify areas of SRF. A custom UNET as described by Ronneberger et al., 2015 (a general training mechanism for a traditional UNET which has been previously implemented), which was trained using data from The Retinal OCT Fluid Challenge (RETOUCH) where Intraretinal fluid (IRF), Subretinal fluid (SRF), Pigment Epithelial Detachment (PED) area, has been manually annotated by multiple observers [19,20]. A three-channel label mask for each OCT scan image was created such that each annotated type was represented in a separate channel of a three channel RGB image. The model was trained on a Windows PC with an 8Gb NVIDIA GTX 1080 graphics card. It was trained for 100 epochs using the UNET with a fixed image size of 512x512, 32 filters and a dropout of 0.2, and only horizontal flipping was randomly augmented. The training data set was separated approximately 2/3 (N = 4,624) for training and 1/3 (N = 2,312) for validation. The image size of each of the SRF scan images was 497 × 495 pixels. Next, the model was tested on a 1/3 validation set. A precision–recall curve (Suppl Figure S2) was constructed for assessing the presence of SRF with the UNET SRF CNN. The OCT SRF CNN was found to have 89% precision, 72% recall, an F1 score of 0.80, and an AUC of 0.91. The SRF CNN was then applied to the 20,630 OCT image from the dataset of 29 Eyes.

Using the OCT SRF CNN, the presence of SRF at 6-monthly periods was used to classify eyes into those with newly developed wet AMD. To allow for the uneven number of OCT scans within 6-month intervals and between eyes, an average area of SRF was computed per interval. New SRF was defined as eyes with SRF on OCT that were classified as SRF-negative in the preceding 6-month time period. The conversion rate recorded in Supplementary Table 4 the cumulative number of eyes with new SRF at any point compared to baseline. For any 6-month time-interval, SRF amount for each eye was taken to be the normalized sum of the detected SRF pixels for all the OCT scans for a specific time point for the whole eye. The accumulated SRF was the volume of SRF per OCT scan, averaged by the number of OCT scans in that 6-month period.

### 2.3. Alignment OCT and DARC and analysis

**Figure 1.**
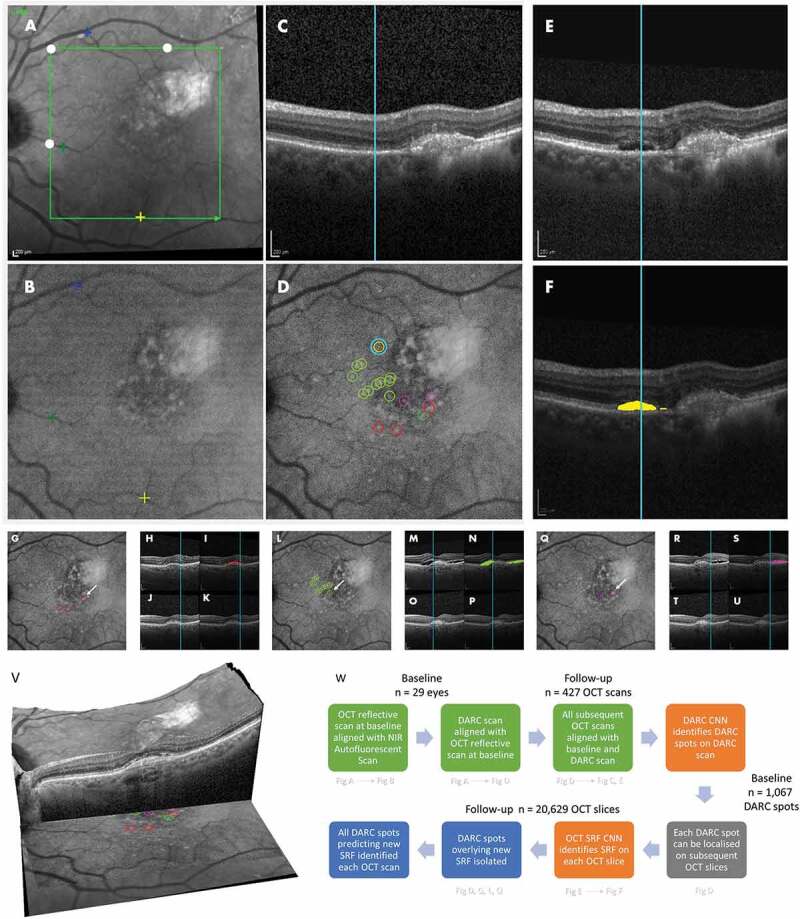
**DARC CNN System predicts new Subretinal Fluid (wet AMD) in OCT at 36 months**. *Alignment application*: for each eye, the reflective OCT localization map (A) is aligned to the baseline autofluorescent image captured with confocal Scanning Laser Ophthalmoscope (cSLO) (B) and the OCT scan (C), utilizing a bespoke application involving automatic landmarks defining OCT areas in serial scans ((filled white circles) and the manual placement of fiducial markers (yellow and blue crosses, A, B). The DARC image (D) is taken 240 minutes after intravenous Anx776 is administered with the cSLO, and is automatically aligned to the baseline autofluorescent image (B) which is now also aligned with the OCT images (A, C). Corresponding points in the DARC image can then be located in the OCT scan (C) using the cyan vertical line, allowing identification of all cross-sectional structures in the OCT image at that vertical location. Hence, in this example, at baseline, the location of the cyan encircled yellow DARC spot (D), can be recorded by x,y coordinates in all the retinal layers of the baseline OCT scan (C). This can be done for all DARC spots, with corresponding points located in all follow-up OCT scans. *DARC spot localization*: DARC spots are automatically detected using a previously described CNN-aided algorithm^3^. The cyan encircled yellow DARC spot (D), is located on the baseline (C) and aligned to the follow-up OCT scan at 36 months (E). The presence of new subretinal fluid (SRF) is automatically identified by a SRF CNN and outlined in the annotated image at 36 months (purple area, F). This shows the cyan encircled yellow DARC spot (D) is predictive of new SRF 36 months later (E, F). *Unique DARC spot prediction*: Each individual DARC spot seen at baseline can be assessed in relation to the series of OCT scans performed at follow-up. Unique DARC spots associated with SRF (D) are encircled with different colors representing different time-points; including: 12–18 months (red, D, G), 18–24 months (light green, D, L), 24–30 months (purple, D, Q) and 30–36 months (yellow, D). These are shown with corresponding OCT scans at follow-up original (H, M, R) and baseline (J, O, T) with SRF CNN-defined newly formed SRF with the same color-coding showing annotated new SRF areas (I, N, S) compared to baseline (K, P, U). The cyan line in each OCT scan highlights the vertical cross-section through the indicated DARC spot (white arrows; G, L, Q). *Alignment of slices from OCT volume scan example and DARC image*. (V) The OCT volume scan made up of 50 OCT slices at baseline is shown with DARC spot localization to summarize the method. *Process flowchart* (W) Each step referring to individual figures (A-U) are outlined in flowchart, which also quantifies the images analyzed in study

DARC images of each eye were aligned with the corresponding OCT series of scans, allowing for the location of a DARC spot to be identified on the corresponding OCT scan at subsequent follow-up. In order to allow for the location of the identified DARC spots in the cSLO images to be referenced against the location of SRF within the OCT images, the DARC cSLO images were registered with OCT images (see Figure 1). Briefly, baseline autofluorescence and DARC images (240 min) were aligned automatically using an affine transformation followed by a non-rigid B-spline transformation. This was completed using multi-resolution alignment methods within SimpleITK (NumFOCUS, US) and a gradient descent optimizer. The reflectance image of sequential Spectralis OCT scans were next aligned with the registered DARC image, by manual placement of fiducial markers (white closed circles, Figure 1) on the cSLO autofluorescence and OCT reflectance images, then calculating and applying an affine transform. Once all the images were aligned then a location on the 240-min DARC image could be selected with the same location being identified in corresponding OCT image slices.

Analysis at each time point was performed to see if a DARC spot from baseline was associated with the occurrence of wet AMD by its intersection with any area of new SRF on OCT as detected by a CNN, as shown in Figure 1 (A-U).

### 2.4. Rabbit study

A pilot study was also performed on three New Zealand White rabbits to assess DARC using an established model of retinal angiogenesis [21]. All experiments were designed and conducted in accordance with the ARVO Statement for the Use of Animals in Ophthalmic and Vision Research and under protocols approved by the UK. Home Office. Briefly, 2.5 kg rabbits were anesthetized with 50 mg/kg intramuscular injection of ketamine hydrochloride and 10 mg/kg xylazine, respectively. Pupils were dilated with 2.5% phenylephrine and 1% tropicamide. 50 ul containing 1 ug of humanVEGF165 (Sigma, UK) dissolved in 0.1% bovine serum albumin (Sigma UK) was injected intravitreally into the left eye only of each animal. Forty-eight hours later, under general anesthesia, rabbits received intravenous Anx776 (0.2 mg) and 1% sodium fluorescein and underwent DARC and 448 imaging at 40 min using high-resolution ICGA and FFA modes of the HRA-Spectralis. Three masked observers performed manual counts on DARC images.

### 2.5. Main outcome measures & statistical analysis

Statistical analysis was performed using GraphPad Prism (version 8.01), Sklearn (version 0.0), SPSS (IBM SPSS version 25) and Python (version 3.6). Precision–recall curves were constructed with precision, recall, F1 score and area under the curve (AUC), generated for SRF CNN testing. Agreement between clinical and CNN definitions of SRF was assessed with kappa statistics [22]. Graphs were constructed based on eyes remaining free of SRF comparing eyes with a total CNN DARC count above and below 5, and compared using Wilcoxon rank pairing for each time point and simple linear regression statistics. Confusion matrices were constructed to assess the prediction rate of eyes with new SRF using only DARC spots overlying SRF, from which positive predictive value (PPV), specificity, and sensitivity were calculated. Correlation of unique DARC spots overlying SRF on OCT with the total area of accumulated SRF on OCT at each time point was made using Spearman’s correlation coefficient (p < 0.05). Assessment of DARC spots overlying SRF at each OCT time point was performed using violin plots, dividing groups into low and high areas of accumulated SRF. SRF high and low groups were compared using Mann–Whitney tests. Manual DARC counts were analyzed using paired t-test comparing treated human-VEGF left to untreated right eyes of rabbits.

## 3. Results

### 3.1. Patient demographics

Nineteen AMD patients were enrolled in the Phase 2 DARC clinical trial at the Western Eye Hospital, ICHNT, London, with baseline demographics and follow-up outlined in Supplementary Tables 1 and 2 according to the detailed exclusion and inclusion criteria in Supplementary Table 3. Of the original 31 eyes assessed with DARC, OCT follow-up was available for 29 eyes followed up regularly with OCT scans for up to 36 months after DARC was performed. The mean number of OCT scans per eye per 6-month interval ranged from 1.70 at 30–36 months to 2.59 at 12–18 months. As patients continued treatment according to the standard of care, the mean number of anti-VEGF intravitreal injections received varied from 2.0 at 30–36 months to 3.61 at 0–6 months. The breakdown of the images analyzed is shown in the ‘Consort’ diagram in Supplementary Figure S1, and the analysis process is summarized in Figure 1.

### 3.2. SRF classification 

From the 29 eyes with available OCT follow-up, 20,629 OCT image slices were analyzed for SRF by the CNN in this study, as each of the 427 OCT scans included consisted of up to 50 separate OCT images per scan.

The occurrence of new SRF at each time period is shown in Table 1 and Supplementary Table 4, with conversion rates of 7.4%, 11.5%, 26.1%, 26.1%, 29.2%, and 33.3% at 6, 12, 18, 24, 30, and 36 months, respectively, using only the definition of OCT CNN SRF. Eyes were classified as ‘converted’ only if they showed new SRF for the first time.

Details of individual eyes according to baseline diagnosis are shown in Supplementary Tables 5 and 6. Of the 11 eyes clinically diagnosed as having active CNN at baseline, the OCT CNN showed the presence of SRF in 8 at baseline. The remaining three eyes with an entry diagnosis of active CNV went on to convert at 6, 18, and 18 months, respectively. Agreement at baseline between the clinical diagnosis of SRF and the OCT CNN identification of SRF was found to be substantial with a kappa value of 0.627 (p = 0.001). One eye with previous CNV and another with late AMD were found to have SRF at baseline using the OCT SRF CNN definition. In all seven eyes were found to convert to new wet AMD over 36 months. Conversion to wet AMD was seen in one eye at 18 months with a baseline diagnosis of previous AMD. Two eyes with early AMD were found to convert to wet AMD at 12 and 30 months, respectively, and an eye with late AMD converted to wet AMD at 6 months.

### 3.3. DARC and SRF formation 

**Figure 2.**
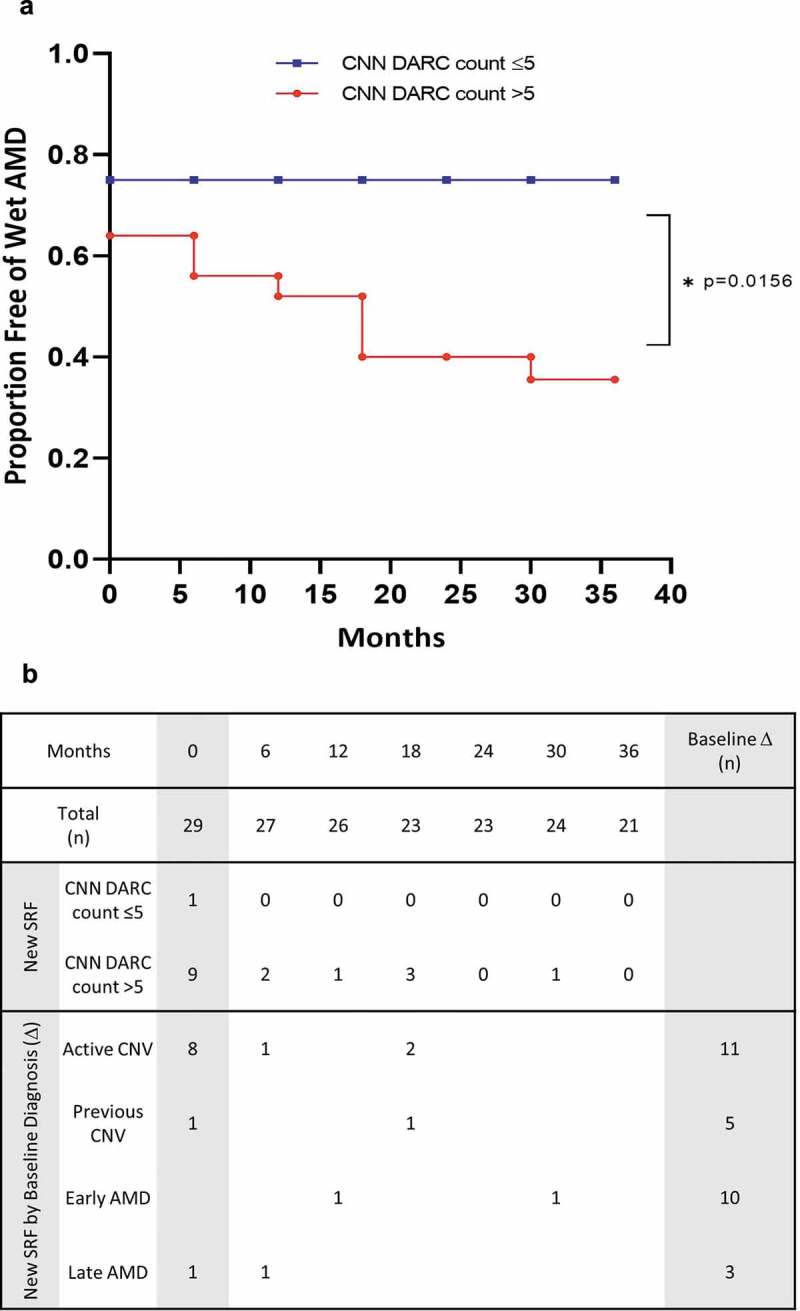
**A CNN DARC Count >5 is associated with risk of developing SRF (wet AMD) in OCT over 36 months**. (A) Plots showing the proportion of eyes remaining free of SRF (free of wet AMD) over 36 months, grouped by the CNN DARC count > 5 (red) or ≤5 (blue). Eyes with a CNN DARC count > 5 have a significantly increased level of developing SRF (Wilcoxon rank, p = 0.0156) compared to those with a CNN DARC count ≤5. (B) Table showing a breakdown of the conversion eyes by baseline diagnosis. All 7 eyes which went on to develop new SRF had CNN DARC counts >5, and 16 of the 17 eyes with either active CNV at baseline (n = 10) or which converted on follow-up (n = 7), had a CNN DARC count >5

Figure 2A shows the proportion of eyes remaining free of SRF (free of wet AMD) at each 6-month time period, and grouped by the CNN DARC count threshold of 5. Comparison between the groups showed that eyes with a CNN DARC count >5 showed a significantly increased level of developing SRF (Wilcoxon rank, p = 0.0156) compared to those with a CNN DARC count ≤5. Linear regressions of the same data were also performed and the slopes of the regression estimates were highly statistically significantly different (p < 0.0001, R^2^ = 0.92 DARC CNN>5 compared to R^2^ = 1.0 DARC count ≤5), indicating a ‘time *group’ effect, that is, that the progress of time affects the group of patients with a high CNN DARC count significantly differently than those with a low count. Observation of the fit line and the data points suggests that the greater hazards occur in the first 16 months, with hazard being relatively flat after that whereas a CNN DARC count > 5 was significantly associated with increased SRF-free survival.

A breakdown of the conversion eyes by baseline diagnosis is shown in Figure 2B. All seven eyes which went on to develop new SRF had CNN DARC counts >5. As shown in Supplementary Table 6, 16 of the 17 eyes with either active CNV at baseline (n = 10) or which converted on follow-up (n = 7), had a CNN DARC count >5.

### 3.4. Unique DARC spots predicting SRF

Both auto-fluorescent and DARC spots overlying SRF in the baseline OCT were excluded from a further analysis, where only the first occurrence of individual DARC spots overlying new SRF (termed ‘unique DARC spot’) were included to avoid repeat counting. At a specific time-point, if an eye showed any unique DARC spots overlying new SRF on OCT, it was classified as true positive; an eye with SRF but no unique DARC spots was false negative; an eye with no SRF but with unique DARC spots was false positive; and eyes with no SRF or DARC spots were true negative. Supplementary Figure S3 shows confusion matrices constructed for each six monthly follow-up period from 6 to 36 months after baseline. Each confusion matrix shows the prediction decision of DARC CNN by eye at each time interval.

Table 1 summarizes the results of the confusion matrices, showing the ability of unique DARC spots to predict eyes with new SRF activity. Hence, at 6 months, the system had a specificity of 90%, a sensitivity of 83%, a PPV of 71%, and an NPV of 95%. The PPV of the DARC DL system is over 70% at all time points, reaching a peak of 86% at 30 months. The specificity ranges from 79% to 90% with sensitivities above 80%. Supplementary Figures S4 to S7 provide further examples of eyes with DARC predicting new SRF.

### 3.5. DARC correlation with SRF size

**Figure 3.**
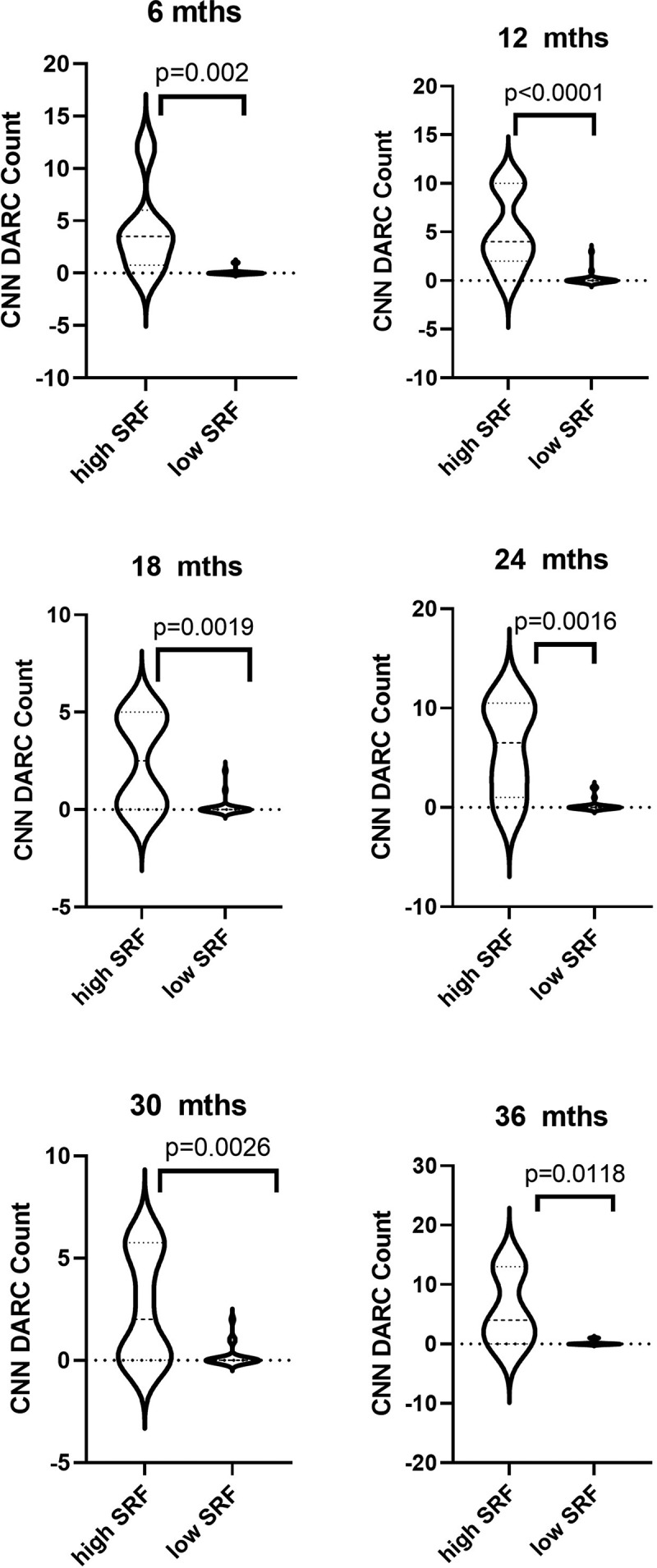
**CNN DARC counts significantly increased in eyes with large areas of SRF accumulation**. Violin plots illustrating the distribution of data of unique DARC spots overlying SRF on OCT with the total area of accumulated SRF on OCT at each time point. To detect CNV lesions on the OCT, a CNN was used to identify areas of SRF, trained using data from The Retinal OCT Fluid Challenge (RETOUCH). To allow for the uneven number of OCT scans within 6-month intervals and between eyes, an average area of SRF was computed per interval. Eyes were divided into ‘high SRF’ and ‘low SRF’ using a threshold of 3000um^2^. Interestingly, a unique DARC count greater than 5 in all eyes, at all time points, is are associated with large SRF accumulation. All p-values indicate level of Mann-Whitney’s significance. Horizontal lines indicate medians and interquartile ranges with minimum and maximum points

The relationship of the DARC count with the magnitude of SRF accumulation amount for each eye at each time-point was next evaluated. Eyes were divided into ‘high SRF’ and ‘low SRF’ using a threshold of 2000 pixels at each time point. Figure 3 shows that DARC counts overlying SRF are significantly (p = 0.002, <0.0001, 0.0019, 0.0016, 0.0026 and 0.0118 at 6, 12, 18, 24, 30 and 36 months, respectively) increased in eyes with large areas of SRF accumulation at each time points. Hence, the greater the SRF, the higher the DARC count, with all eyes having a unique DARC count greater than 5 associated with large SRF accumulation. Indeed, the DARC spots overlying SRF at each time point was positively correlated with the OCT SRF area at 6 (Spearman’s r = 0.65, p = 0.0001), 12 (r = 0.72, p < 0.0001), 18 (r = 0.60, p = 0.0006), 24 (r = 0.57, p = 0.0012), 30 (r = 0.67, p < 0.0001), and 36 (r = 0.59, p = 0.0008) months.

### 3.6. DARC identifying angiogenesis in rabbit model

**Figure 4.**
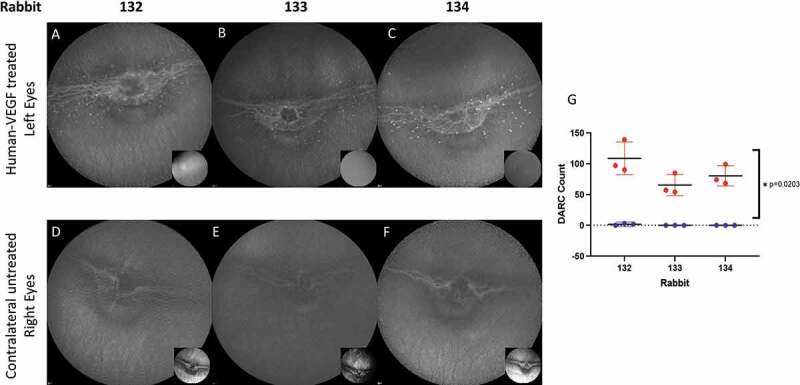
**DARC identifies earliest changes of endothelial activity in a rabbit model of angiogenesis**. A rabbit model of angiogenesis was created by intravitreal administration of 1 ug in 50 ul of human VEGF (hVEGF165 – Peprotech, London UK) to the left eyes only of 3 rabbits. 2 and 4 days later intravenous ANX776 (0.2 mg) and 1% sodium fluorescein was given. Both eyes of rabbits were imaged using the ICGA and FFA settings of cSLO. DARC spots (identified as white spots which are ANX776 positive-labeled) are clearly visible in the left (A-C) hVEGF treated eyes compared to untreated right eyes (D-F). DARC counts were obtained by three masked manual observers. (G) Individual points represent separate manual observations of left and right eyes. All hVEGF eyes showed positive DARC staining compared to untreated, contralateral eyes. There was a significant difference (p = 0.0203) between DARC counts in treated compared to control eyes. Inserts (A-F) show fluorescein angiography 4 days after treatment with hVEGF. All treated eyes went on to show fluorescein leakage at 4 days, (inserts A-C) compared to control (inserts D-F)

All rabbit eyes treated with hVEGF were found to have DARC positive-staining compared to the untreated contralateral eye 2 days after treatment. Figure 4 shows DARC images taken of both eyes of each animal, with DARC spots clearly visible in the left (A-C) hVEGF treated eyes compared to untreated right eyes (D-F), with a significant increase (p = 0.0203, G) in treated eyes. All treated eyes went on to show fluorescein leakage at 4 days, as shown in the inserts A-C compared to control (inserts D-F).

## 4. Discussion

An increasingly recognized unmet need is the prediction of new CNV in patients with AMD so that visual impairment can be avoided through the early intervention of effective therapies [10]–[13]. The study demonstrates for the first time the application of DARC to wet AMD. Using the recently described CNN algorithm, DARC was able to predict the development of new SRF over a 36-month period of follow-up, with PPV, specificities, and sensitivities above 70%, with a CNN DARC count greater than 5 significantly associated with SRF formation. Moreover, the magnitude of SRF was related to that of the DARC count, and this is attributed to the mechanism by which DARC identifies an early endothelial angiogenic activity, as seen for the first time *in vivo* in a rabbit model of hVEGF-induced angiogenesis/leakage. This is the first time DARC technology has been reported in a disease other than glaucoma and suggests its broader applicability as a biomarker of retinal activity [17] [18].

The AMD patients in this study were part of the Phase 2 DARC clinical trial with eyes with both dry and wet AMD, based on a clinical definition. Recently, it has been shown in two separate studies that clinical identification of SRF on OCTs is less accurate than automated algorithms, including a prospective study with data from AREDs by Keenan et al. with measurements of sensitivity being 0.583 by specialists compared to 0.940 by the AI system when detecting SRF [14]. Similarly, Yim et al. used as a ground truth a ‘clinical expert benchmark’ when trying to predict wet AMD from OCT scans, and noted that the agreement between clinicians was low (Kappa between 0.143 and 0.335), and showed an AI system perform better than the clinical experts [10]. For this reason, in this post-hoc study, we used an objective measure of OCT subretinal fluid (SRF) accumulation to define new SRF and wet AMD conversion, and not a clinical definition.

The DARC CNN-aided algorithm used in this study was identical to that published earlier this year [17]. Great efforts were taken in the training of the CNN for DARC spot identification. Specifically, to avoid over-training, training was performed only on DARC spots from 50% of the control cohort of eyes in the Phase 2 trial. The remaining 50% was used for validation/testing with the final testing performed on the glaucoma cohort. It is important to highlight that there were no modifications at all in the CNN DARC spot identification in the present analysis reported here.

A central factor in this study is the use of a CNN to define SRF in OCT scans as opposed to that identified by clinicians. Manually annotating SRF in over 20,000 OCT slices would have been an extremely time-consuming and onerous task for any reader. Furthermore, the application of AI algorithms for automatically quantifying SRF is increasingly recognized [23] [14]. The OCT SRF CNN applied in this study had 89% precision, 72% recall, an F1 score of 0.80, and an AUC of 0.91, again comparing favorably to published literature. An interesting finding in this study, however, was the level of agreement of the presence of baseline SRF by clinical and CNN detection, which was substantial. It is interesting that the three eyes classified clinically at baseline as having active CNV, yet which had no SRF identified by the CNN, then went on to develop new SRF. This does suggest that the clinical diagnosis was based on features additional to OCT, perhaps consistent with several risk factors identified by previous studies [23–26]. There is also recent evidence comparing retinal specialists’ identification of CNV with reading centers showing agreement of 74.9%, which highlights clinical discrepancies [27]. But the application of AI to detect CNV is important, especially as compared to DL systems, retinal specialists have been shown to have ‘imperfect accuracy in detecting retinal ﬂuid, with low sensitivity’ [14]. Additionally, AI algorithms provide a practical solution, as the clinical assessment is time-consuming and expensive, and automated systems can provide objective, consistent and scalable applications [28].

The conversion rates in our study reflect the formation of new SRF only and do not include intraretinal fluid or pigment epithelial detachment conversion. Additionally, our data were not derived solely from fellow eyes of wet AMD, as detailed in Supplementary Table 4. However, despite differences in sample sizes, our results appear similar to several published rates at 6, 12, 18, 24, 30, and 36 months, being comparable to Fasler [29], Bek [30], Zarranz-Ventura [31], Marques [26] and Banerjee [13]. As we looked at eyes with different types of AMD, and not just fellow eyes of wet AMD patients, it is interesting that our conversion rates are similar, and suggests the risk of conversion is present with any eye with evidence of AMD. We, therefore, believe that despite the small numbers in our study, conversion of eyes to wet AMD is similar to those previously reported.

Although it is not common practice for clinicians to predict CNV using OCT, the benefits of being able to do so have led to the use of AI systems to address this challenge [10]. One of the earliest studies by de Sisternes in 2014 was very encouraging, and several studies from around the world have advanced this area as detailed in Supplementary Table 7 [10,12,13,32,33].

At the time of our analysis, a paper by Yim et al. was published which described the use of AI with OCT to improve predicting the development of CNV or exudative age-related macular degeneration (exAMD) at 6 months by analyzing OCT features [10]. However, despite the novelty and combination of two different DL models, it was interesting that they reported low predictive values; namely, a specificity of 55% at 80% sensitivity, a sensitivity of 34% at 90% specificity. It was also concerning that the authors omitted to discuss their poor positive predictive values (PPV) of 7.3% and 13.2%, respectively, given the importance of the very concept of PPV in a clinical prediction study [34] [35,36]. With the DARC CNN system, it is truly encouraging to see a high PPV compared to other studies outlined in Supplementary Table 7, although we recognize that further improvement could still be achieved.

The improvement in the predictive value of the DARC CNN as opposed to previously reported OCT AI systems is related to its indication of cellular activity. Recently, Li et al. reported that the process of phosphatidylserine exposure on endothelial cells is one of the earliest stages in neovascularization [37]. However, this was a histological study using retina from mice with laser-induced CNV, where phosphatidylserine was shown to be exposed in CNV lesions, and where antibodies targeting phosphatidylserine reduced CNV lesion size. Using an animal model (Figure 4) to validate these findings, we show for the first time in vivo that DARC can identify phosphatidylserine in angiogenic processes. The model clearly demonstrated vascular leakage occurring at 4 days, with DARC activity seen 2 days earlier. Mechanistically, this can be explained by the binding of the annexin V biomarker in DARC to exposed phosphatidylserine in endothelial cells in the earliest stages of angiogenesis in CNV lesions. In human eyes, the DARC signal also indicates and predicts new vessel formation, heralding leakage and retinal fluid formation, as borne out by single DARC spots predicting new areas of SRF. This is also reflected by a CNN DARC count >5 being associated with either existing or future SRF activity. The level of DARC activity indicates accumulation, with the greater the SRF, the higher the DARC count, with all eyes having a unique DARC count greater than 5 associated with large SRF accumulation.

An issue highlighted by the use of AI is that it often does not provide any explanation of what mechanism of action might be obtained from the ‘black box’ result. The concept of AI as a magic black box is coming under increasing criticism, with the lack of causal inference making predictions uninterpretable and frequently irreproducible on a new data set. Indeed, it has been suggested that AI systems should identify abnormal areas contributing to their prediction through the use of ‘saliency maps’ [38]. This is compounded by the acknowledgment that the prediction of wet AMD using OCT is not something routinely done clinically [10]. In contrast, using a non-OCT imaging technology, here we describe the causal relationship by which DARC is directly associated with the magnitude of SRF accumulation.

Although the results in this study are encouraging, we recognize that it is only based on a small number of patients and is a post hoc analysis. We would hope to further validate our results in the future in perspective and larger clinical trials, with strict protocols with respect to patient management. We believe that this should lead to increased sensitivities and specificities. In addition, the algorithm could be further refined, as we have recently discussed in the DARC CNN publication [17]. Furthermore, there was no strict requirement for patients to have FFA for a definitive diagnosis of CNV, which would have highlighted the presence of active leakage and membrane formation, and also enable classification of Type 1, 2, and 3 CNVs [39,40]. It would be very interesting to see if DARC can identify these abnormalities, including in the presence of scar formation and disorganized retinal architecture.

However, the major advantage of DARC, as opposed to OCT, is it provides a cellular activity readout. The ability to identify endothelial cells in the initial stages of angiogenesis is key we believe in predicting future SRF accumulation. Given the importance of early identification of new wet AMD disease, any inaccuracies in diagnosis could lead to treatment delays and ensuing vision loss; hence, a high PPV, a validated ground truth, and a mechanistic explanation are desirable in AI-predictive systems, especially if they are to be clinically useful.

## 5. Conclusion

OCT provides macroscopic and structural information. In comparison, DARC detects cellular changes that identify early endothelial neovascular activity. In light of our results, we suggest that the integration of DARC with CNN systems can give more accurate results in predicting wet AMD than a purely based OCT AI system, with important clinical and health-economic implications in the management of the preventable sight-threatening disease.

## Supplementary Material

Supplemental Material

## Trial Registration

ISRCTN10751859
