# Supplementary material for: Predicting wet age-related macular degeneration (AMD) using DARC (detecting apoptosing retinal cells) AI (artificial intelligence) technology
Source: Expert Rev Mol Diagn. 2020 Dec 28;21(1):109–18. doi: 10.1080/14737159.2020.1865806 (PMC8011474; doi:10.1080/14737159.2020.1865806)
Supplement: Supplemental Material [file IERO_A_1865806_SM4022.docx]

Supplementary information

**Predicting wet age-related macular degeneration (AMD) using DARC (Detecting Apoptosing Retinal Cells) AI (artificial intelligence) technology**

Table of Contents

[Supplementary Table 1: Demographics, Baseline characteristics. 2](#_Toc50666058)

[Supplementary Table 2: Follow up summary 3](#_Toc50666059)

[Supplementary Table 3: Inclusion and exclusion criteria 4](#_Toc50666060)

[Supplementary Figure S1: Consort Diagram 5](#_Toc50666061)

[Supplementary Figure S2: Precision Recall Curve of SRF CNN algorithm 6](#_Toc50666062)

[Supplementary Table 4: Conversion rates of New SRF Comparison ^1^ ^2^ ^3^ ^4^ ^5^ ^6^ ^7^ ^8^ ^9^ ^10^ 7](#_Toc50666063)

[Supplementary Table 5: Summary of Eyes by Baseline Diagnosis, Injections and Conversion 7](#_Toc50666064)

[Supplementary Table 6: Breakdown of Eyes by Baseline Diagnosis, Injections and Conversion 9](#_Toc50666065)

[Supplementary Figure S3: Confusion matrices 10](#_Toc50666066)

[Supplementary Figure S4: DARC and OCT image alignment application 11](#_Toc50666067)

[Supplementary Figure S5: Typical example of aligned DARC and OCT images in patient with SRF showing prediction of OCT SRF by DARC spots. 12](#_Toc50666068)

[Supplementary Figure S6: Example of DARC predicting wet AMD 13](#_Toc50666069)

[Supplementary Figure S7: Images of Individual Eyes with Highlighted DARC spots Corresponding with New SRF formation 14](#_Toc50666070)

[Supplementary Table 7: Prediction Values of CNV by AI Systems^10^ ^11^ ^9^ ^12^ ^13^ 23](#_Toc50666071)

[References 24](#_Toc50666072)

## Supplementary Table 1: Demographics, Baseline characteristics.

| **Baseline Characteristic** | | | **Patients/Eye** | |
| --- | --- | --- | --- | --- |
| Age | Mean years (SD) | | 82.89 | (5.84) |
| Gender | Males n° (%) | | 11 | (57.89) |
|  | Females n° (%) | | 8 | (42.11) |
|  | Total | | 19 |  |
| Ethnicity | Caucasian (%) | | 15 | (78.95) |
|  | Black (%) | | 0 |  |
|  | Hispanic (%) | | 0 |  |
|  | Asian (%) | | 4 | (21.05) |
|  | Other (%) | | 0 |  |
| Vital signs | Mean Blood Pressure | Systolic, mmHg (SD) | 142 | (17.5) |
|  |  | Diastolic, mmHg (SD) | 71 | (9.6) |
|  | Heart Rate | Beats/min (SD) | 65 | (9.7) |
|  | Respiratory Rate | Resp/min (SD) | 17 | (1.4) |
|  | Height | Metres (SD) | 1.62 | (0.1) |
|  | Weight | Kg (SD) | 65.8 | (9.9) |
| Study Eye | Right (%) |  | 3 | (15.79) |
|  | Left (%) |  | 4 | (21.05) |
|  | Both (%) |  | 12 | (63.16) |
|  | Total |  | 31 | (100) |
| BCVA | Affected eye | Logmar Mean (SD) | 0.39 | (0.41) |
|  | Unaffected eye |  | 0.27 | (0.4) |
| IOP | Affected eye | mmHg (SD) | 13.5 | (3.55) |
|  | Unaffected eye |  | 13.7 | (2.88) |

## Supplementary Table 2: Follow up summary

| **Intervention** | | Mean/eye | (SD) |
| --- | --- | --- | --- |
|  |  |  |  |
| Study Eyes | Total n=29 |  |  |
| OCT scans | 0-6 months | 2.48 | (1.67) |
|  | 6-12 months | 2.45 | (1.86) |
|  | 12-18 months | 2.59 | (1.52) |
|  | 18-24 months | 2.37 | (1.36) |
|  | 24-30 months | 2.15 | (1.38) |
|  | 30-36 months | 1.70 | (1.32) |
|  |  |  |  |
| Anti-VEGF Injections |  |  |  |
|  | Before DARC | 15.58 | (13.90) |
|  | 0-6 months | 3.61 | (0.60) |
|  | 6-12 months | 2.94 | (1.00) |
|  | 12-18 months | 2.33 | (1.40) |
|  | 18-24 months | 2.39 | (0.77) |
|  | 24-30 months | 2.25 | (1.29) |
|  | 30-36 months | 2.00 | (0.94) |

## Supplementary Table 3: Inclusion and exclusion criteria

INCLUSION CRITERIA

1. Age ≥ 18 years.
2. Clear optical media in the studied eye.
3. Refractive error not higher than spherical equivalent of 10 D and best corrected visual acuity equal to 6/24 or better at qualification.
4. Women of childbearing potential identified as not pregnant and have consented to complete a pregnancy test.
5. Subjects who have capacity to consent, have personally signed and dated the informed consent document indicating that they have been informed of all pertinent aspects of the study.

EXCLUSION CRITERIA

1. Presence of severe, unstable or uncontrolled systemic disease.
2. Known intolerance to IMP.
3. Body weight <40kg or >150kg.
4. Inability to comply with the study or follow-up procedures.
5. Any subjects with a known history of clotting diseases (including DVTs), and subjects taking anticoagulants.
6. Ocular surgery within the past 3 months or planned surgery in the study eye, during the course of the trial.
7. Pregnant or lactating, or not using adequate contraception* for the duration of the trial (and 30 days post injection of study drug).
8. Currently being treated for cancer or any other disease likely to adversely affect participation in this study.
9. AIDS / HIV.
10. History of alcoholism or drug addiction.
11. History or active uveitis.
12. History of systemic vasculitis, collagenosis or ongoing treatment of cancer.
13. Evidence of previous retinal vascular disease.
14. Individuals with terminal illness, or mental illness affecting their compliance with the study
15. Any other disease, condition or laboratory abnormality that in the opinion of the CI may increase the risk for the participation or may interfere with the interpretation of study results and in the judgement of the Investigator would make the subject inappropriate for entry into the study.
16. Central corneal thickness <450 pm or >650pm.
17. Currently, or within the last 3 months, enrolled in a clinical trial of an investigational medicinal product.
18. History of retinal laser photocoagulation.
19. Media opacities or retinal pathology or amblyopia significantly limiting visual acuity, visual field test or retinal imaging.
20. Any other condition or pathological process that in the opinion of the investigator would not make the patient suitable for the trial.

## Supplementary Figure S1: Consort Diagram


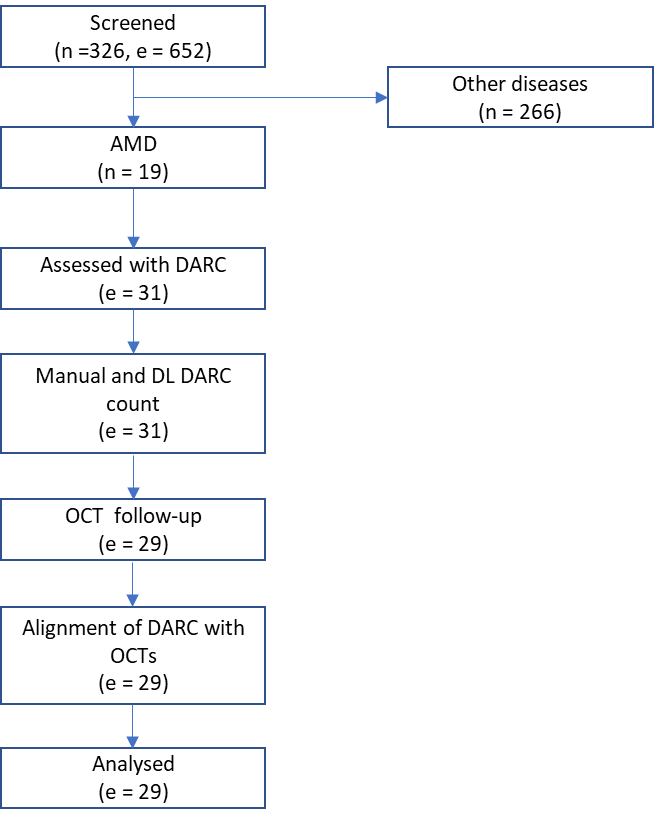


## Supplementary Figure S2: Precision Recall Curve of SRF CNN algorithm


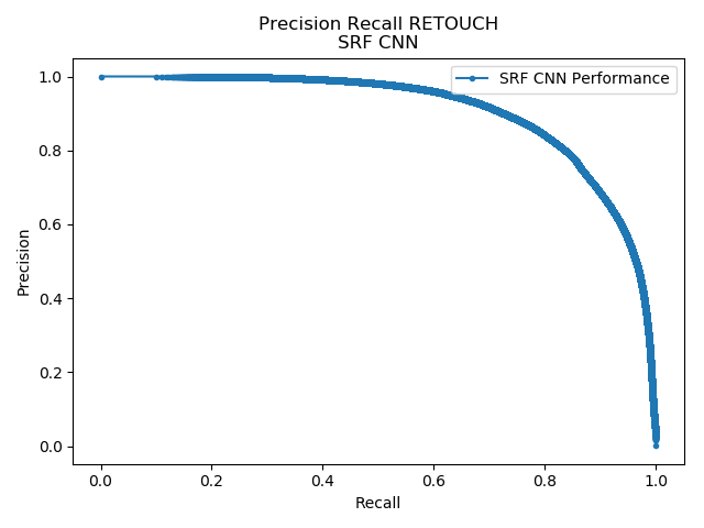

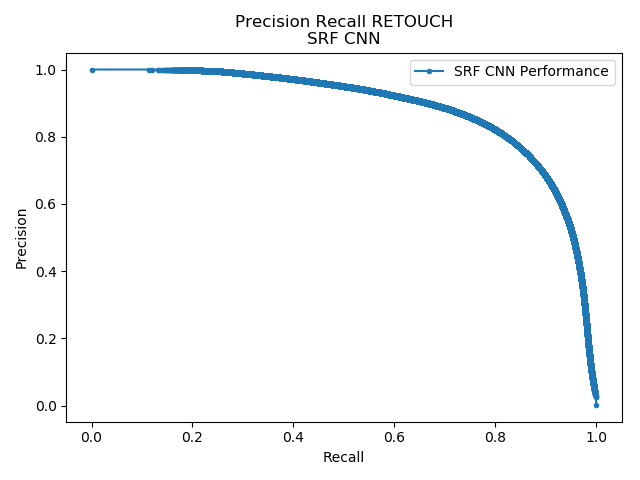


A precision-recall curve was constructed with the UNET CNN (as described by Ronneberger et al. 2015) ^14^, from the The Retinal OCT Fluid Challenge (RETOUCH) ^15^ where Intraretinal fluid (IRF), Subretinal fluid (SRF), Pigment Epithelial Detachment (PED) area have been manually annotated by multiple observers. The precision-recall curve below was constructed for assessing the presence of SRF with the SRF CNN. The model was trained for 100 epochs with approximately 2/3 (N=4,624) of the dataset for training and 1/3 (N=2,312) for validation. The SRF CNN was found to have 89% precision, 72% recall, an F1 score of 0.80 and an AUC of 0.91.

## Supplementary Table 4: Conversion rates of New SRF Comparison ^1^ ^2^ ^3^ ^4^ ^5^ ^6^ ^7^ ^8^ ^9^ ^10^

##
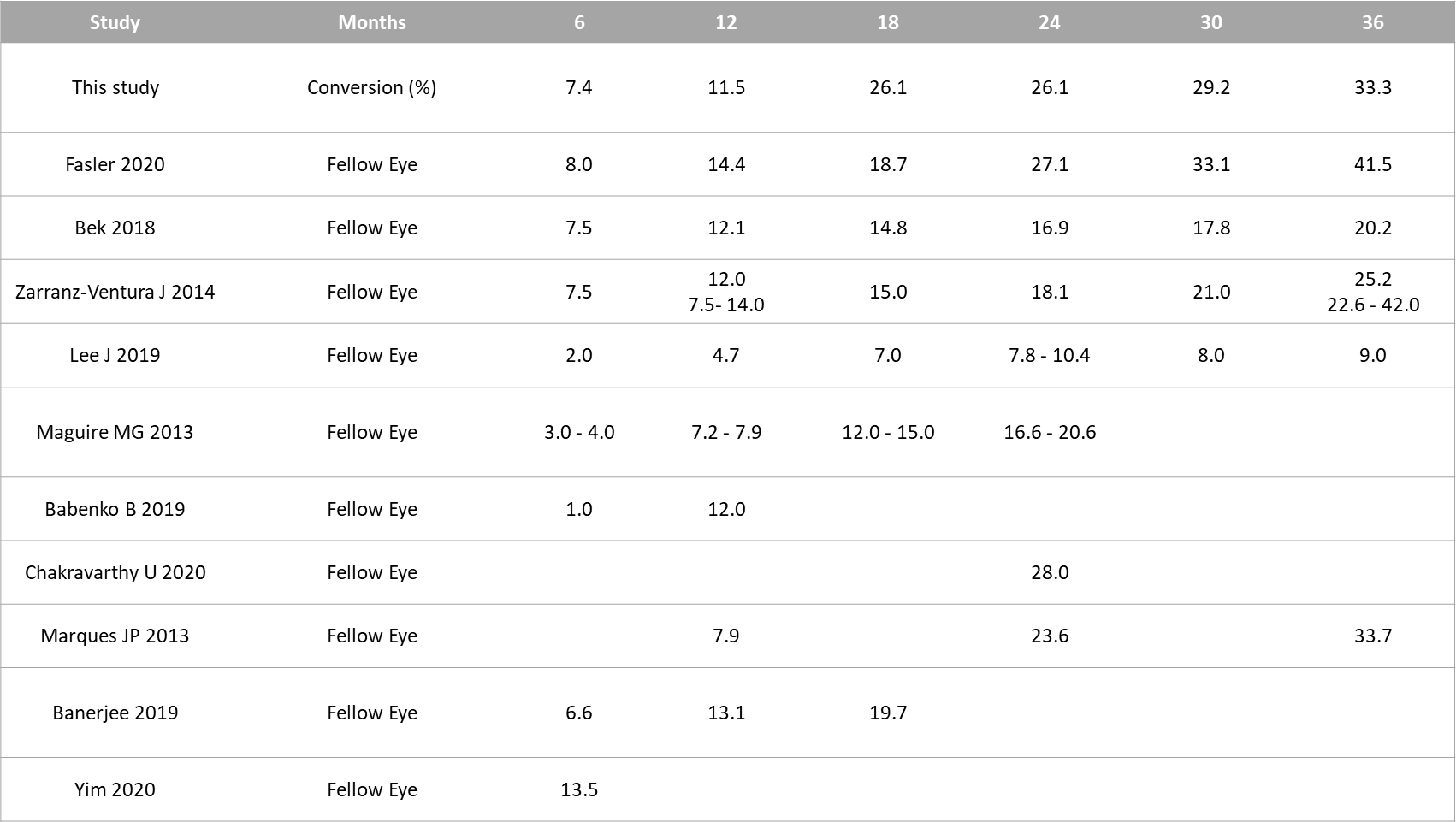
Supplementary Table 5: Summary of Eyes by Baseline Diagnosis, Injections and Conversion

| **Baseline Diagnosis** | **Number of eyes** | **Injections in follow-up** | **OCT CNN SRF**  **Positive Baseline** | **Conversion in follow-up** |
| --- | --- | --- | --- | --- |
| Active CNN | 11 | 11 | 8 | 3 |
| Previous CNN | 5 | 3 | 1 | 1 |
| Early AMD | 10 | 2 | 0 | 2 |
| Late AMD | 3 | 0 | 1 | 1 |
| Total  (n) | 29 | 16 | 10 | 7 |

## Supplementary Table 6: Breakdown of Eyes by Baseline Diagnosis, Injections and Conversion


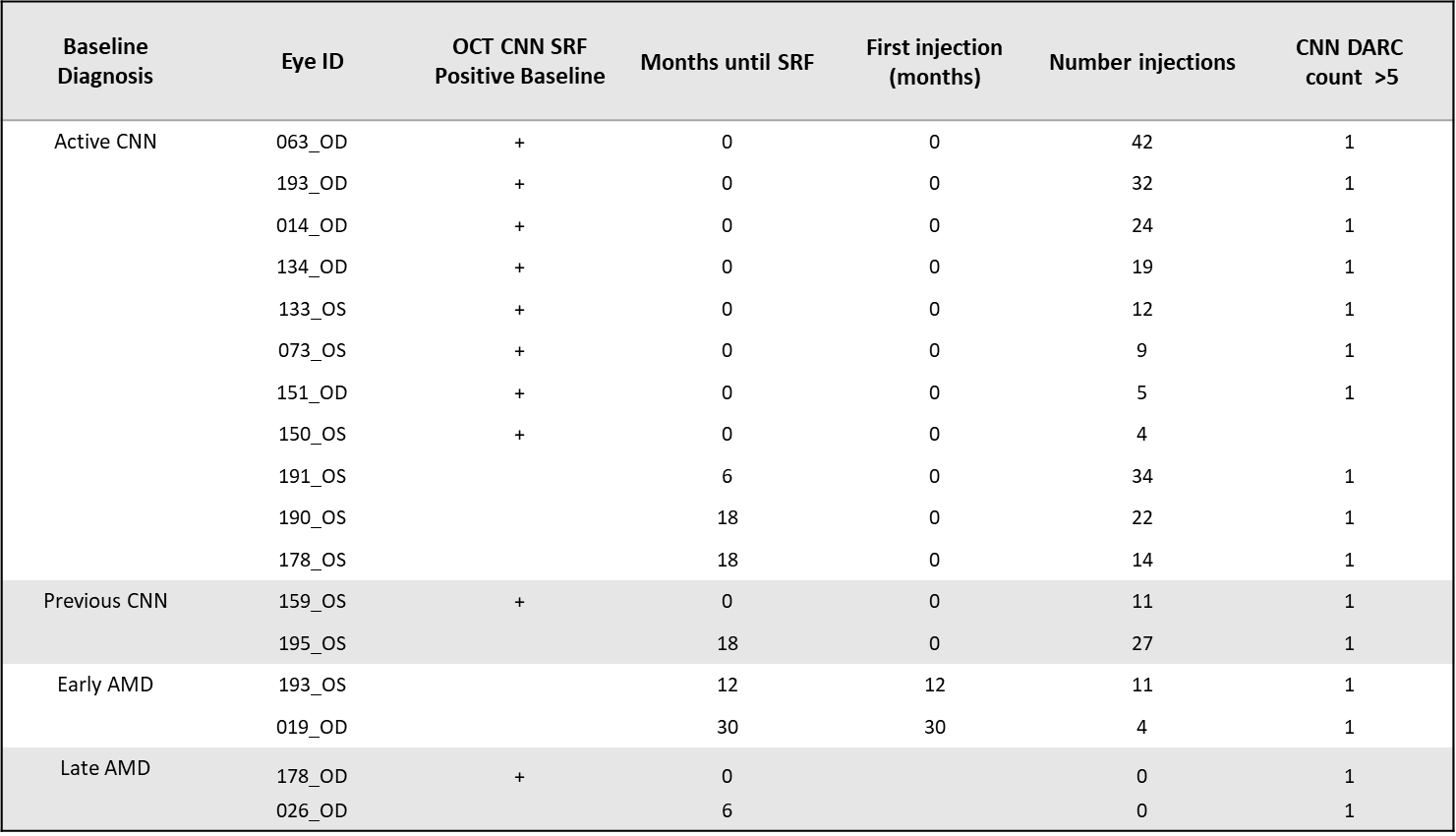


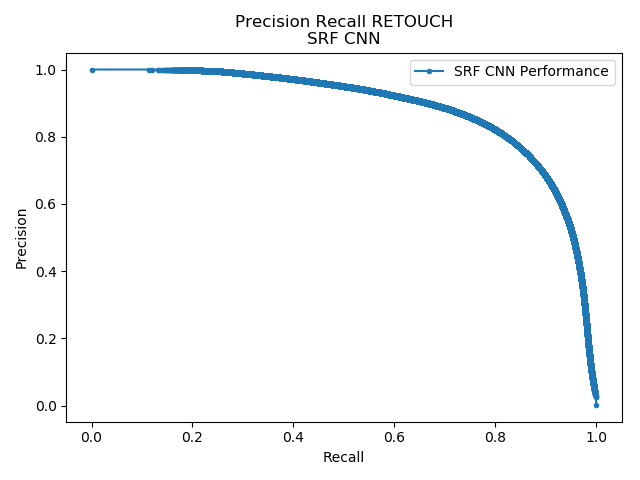


## Supplementary Figure S3: Confusion matrices

|  |  |  |  |  |  |  |  |  |
| --- | --- | --- | --- | --- | --- | --- | --- | --- |
|  | A. | **6 MONTHS** | |  | B. | **12 MONTHS** | |  |
|  |  | **OCT SRF +** | **OCT SRF -** |  |  | **OCT SRF +** | **OCT SRF -** |  |
|  | **DARC DL SRF +** | 5 | 2 |  | **DARC DL SRF +** | 7 | 3 |  |
|  | **DARC DL SRF -** | 1 | 19 |  | **DARC DL SRF -** | 1 | 18 |  |
|  |  |  |  |  |  |  |  |  |
|  | C. | **18 MONTHS** | |  | D. | **24 MONTHS** | |  |
|  |  | **OCT SRF +** | **OCT SRF -** |  |  | **OCT SRF +** | **OCT SRF -** |  |
|  | **DARC DL SRF +** | 9 | 2 |  | **DARC DL SRF +** | 10 | 3 |  |
|  | **DARC DL SRF -** | 2 | 16 |  | **DARC DL SRF -** | 2 | 14 |  |
|  |  |  |  |  |  |  |  |  |
|  | E. | **30 MONTHS** | |  | F. | **36 MONTHS** | |  |
|  |  | **OCT SRF +** | **OCT SRF -** |  |  | **OCT SRF +** | **OCT SRF -** |  |
|  | **DARC DL SRF +** | 12 | 2 |  | **DARC DL SRF +** | 12 | 3 |  |
|  | **DARC DL SRF -** | 3 | 12 |  | **DARC DL SRF -** | 3 | 11 |  |
|  |  |  |  |  |  |  |  |  |
|  |  |  |  |  |  |  |  |  |

Confusion matrices for the prediction decision of DARC DL by eye at 6 (A), 12 (B), 18 (C), 24 (D), 30 (E) and 36 (F) months after DARC. The presence (**OCT SRF+**) or absence (**OCT SRF-**) of subretinal fluid (SRF) on the OCT at each time point was used as the comparator. At each time-point, if an eye showed any unique DARC spots overlying SRF (**DARC DL SRF +**) in an eye that was **OCT SRF +**, it was classified as true positive (green); an eye with **OCT SRF +** but no unique DARC spots (**DARC DL -**) was false negative (blue); an **OCT SRF -** eye but with unique DARC spots (**DARC DL SRF +**) was false positive (red); and eyes with no OCT SRF (**OCT SRF** **-**) or DARC spots (**DARC DL** **SRF** **-**) were true negative (yellow).

## Supplementary Figure S4: DARC and OCT image alignment application


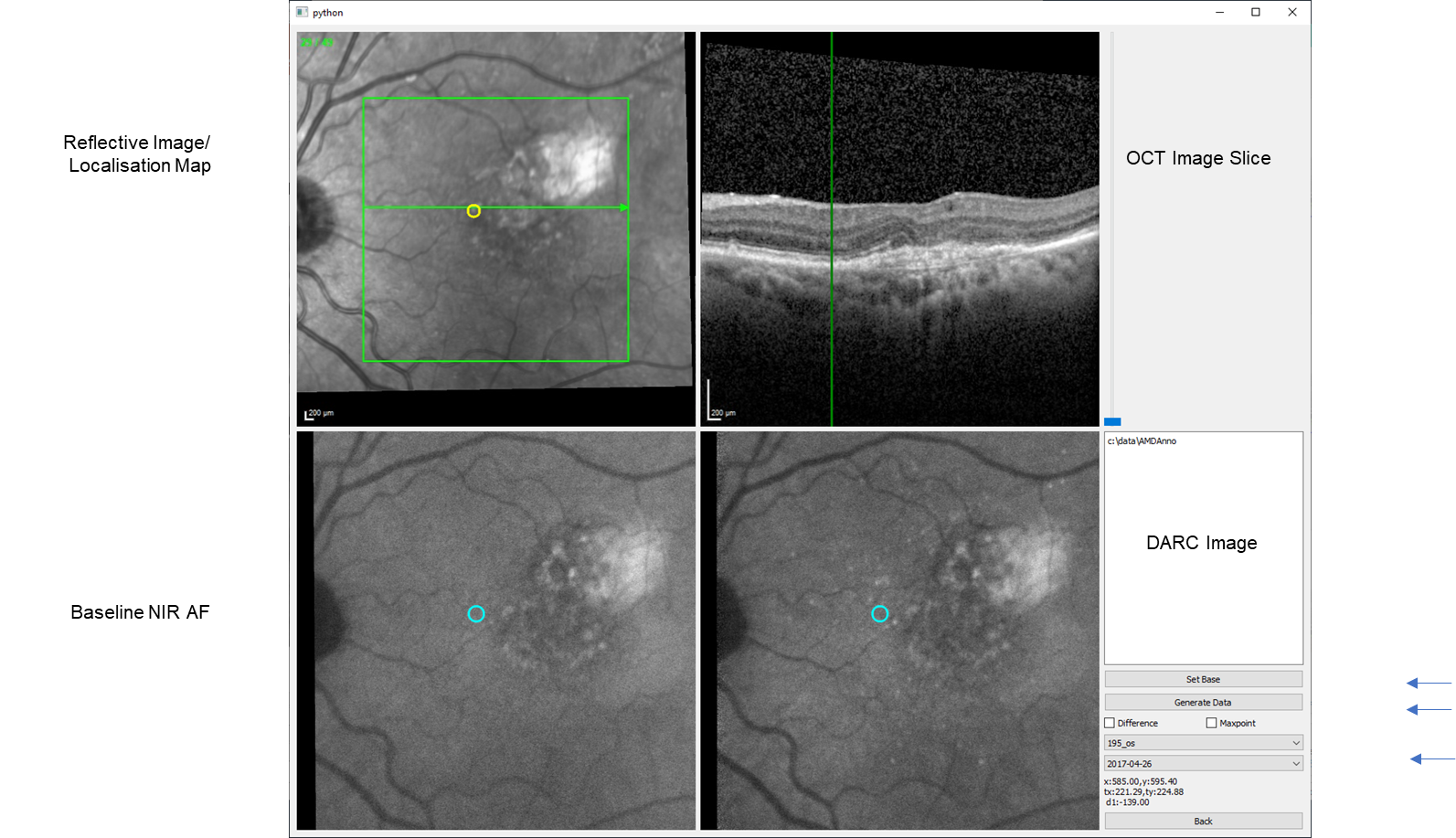


B

A

D

C

The OCT images with localisation map are shown in A and B respectively. A NIR auto fluorescent baseline image is shown in C. The DARC image (D) is taken 240 minutes after iv Anx776 is given.

Serial OCTs were analysed from the same eyes. These were aligned with the DARC images so that localisation of the DARC signal could be performed on the OCT. Each eye and the date of the OCT examination are shown as indicated, with the xy coordinates of identified DARC spot (cyan circle) also, using a bespoke application algorithm.

## Supplementary Figure S5: Typical example of aligned DARC and OCT images in patient with SRF showing prediction of OCT SRF by DARC spots.


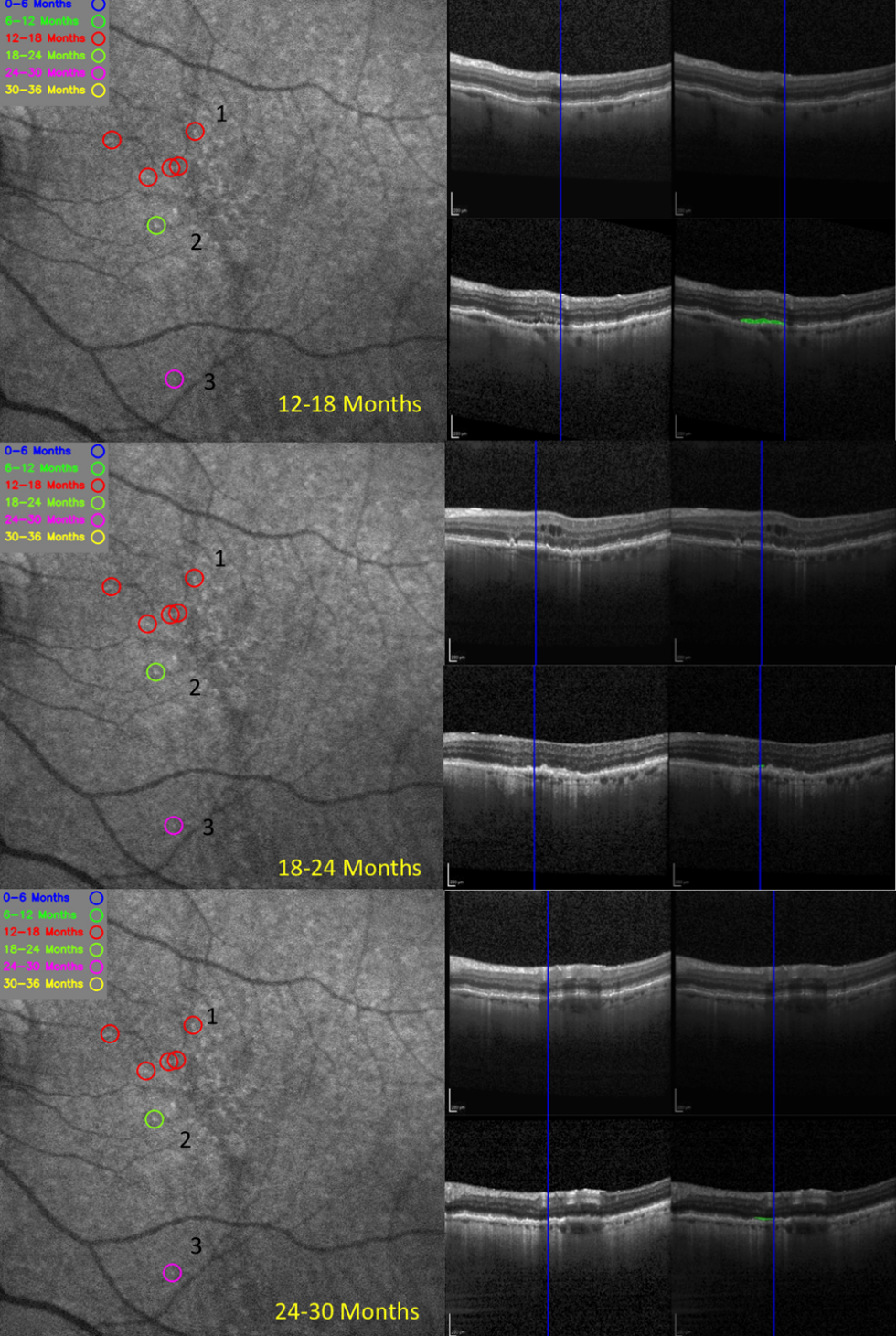


C

A

B

D

E

H

G

F

J

I

M

L

K

O

N

DARC images of the same eye are shown at indicated time points in A, F and K. DARC spots are outlined with circles as detected by a CNN-aided algorithm^7^. The different coloured circles represent the unique DARC spots seen at each time point. B-E, G-J and L-O are OCT images taken at baseline (B-C, G-H and L-M) and corresponding time points after DARC (D-E at 12-18 months, I-J at 18-24 months, N-O at 24-30 months). The dark blue line highlights the cross-section through the indicated DARC spot (yellow arrows at 1, 2 and 3). Original OCTs (B, D, G, I, L, N) are shown alongside images with OCT DL algorithm outlining SRF lesions in green (C, E, H, J, M, O).

## Supplementary Figure S6: Example of DARC predicting wet AMD


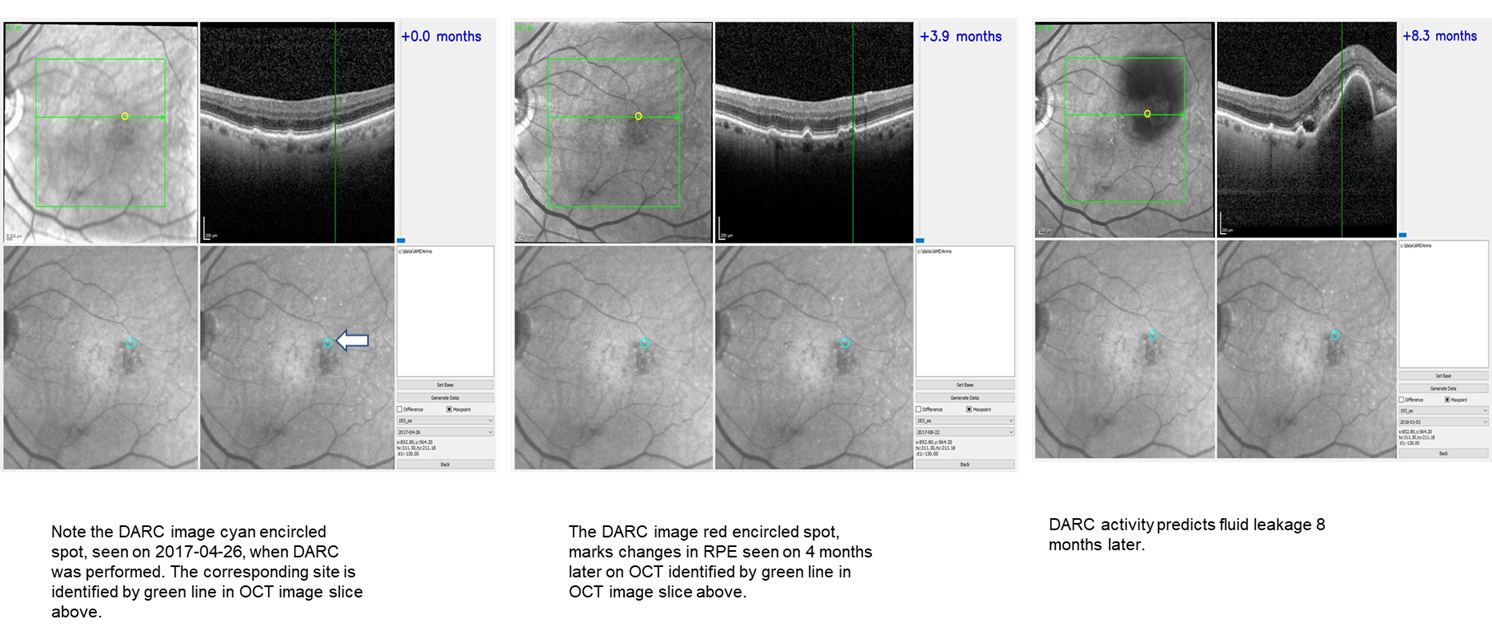


## Supplementary Figure S7: Images of Individual Eyes with Highlighted DARC spots Corresponding with New SRF formation

The following DARC images are from all eyes with DARC spots corresponding to new SRF highlighted by time point.


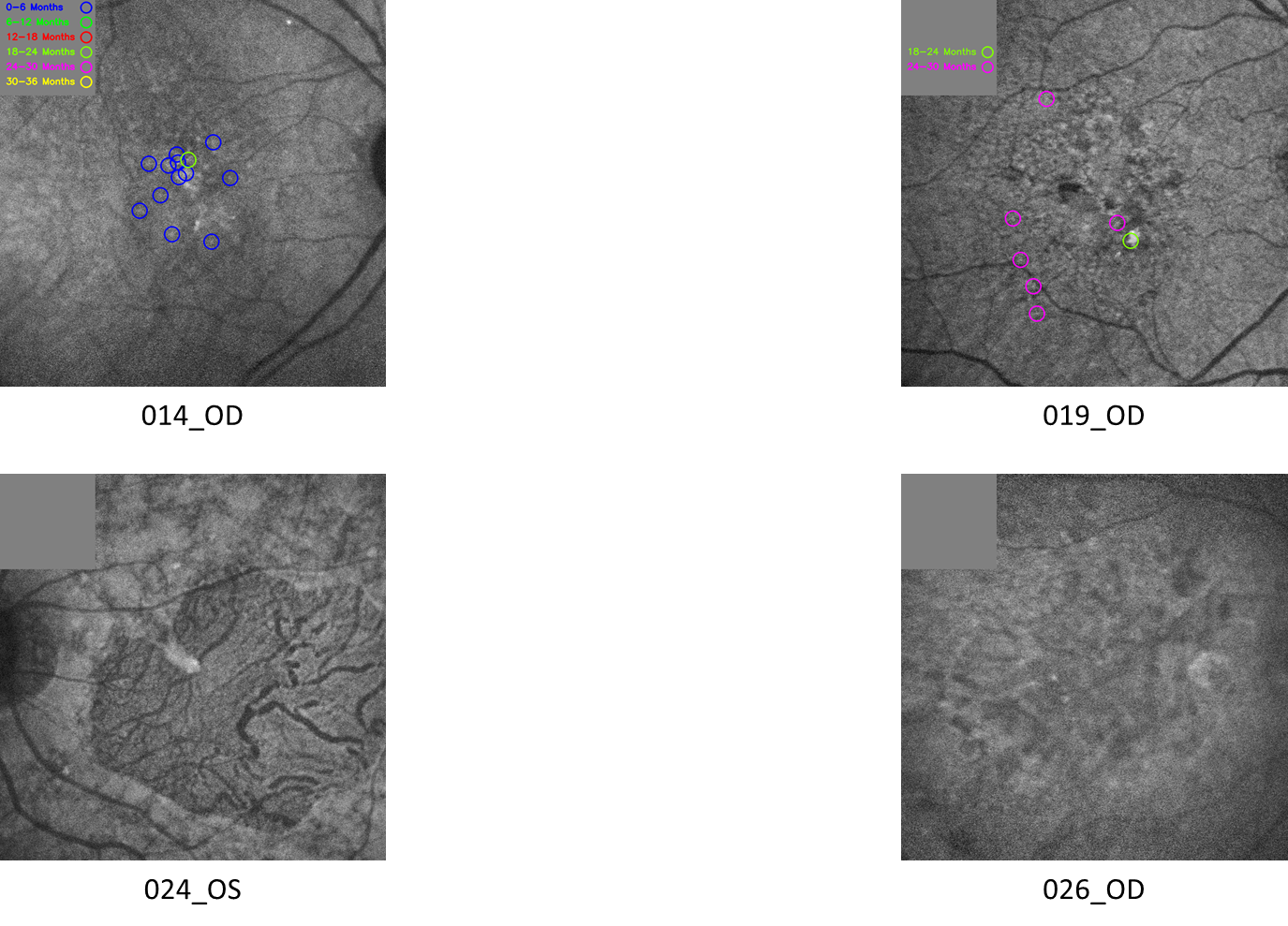


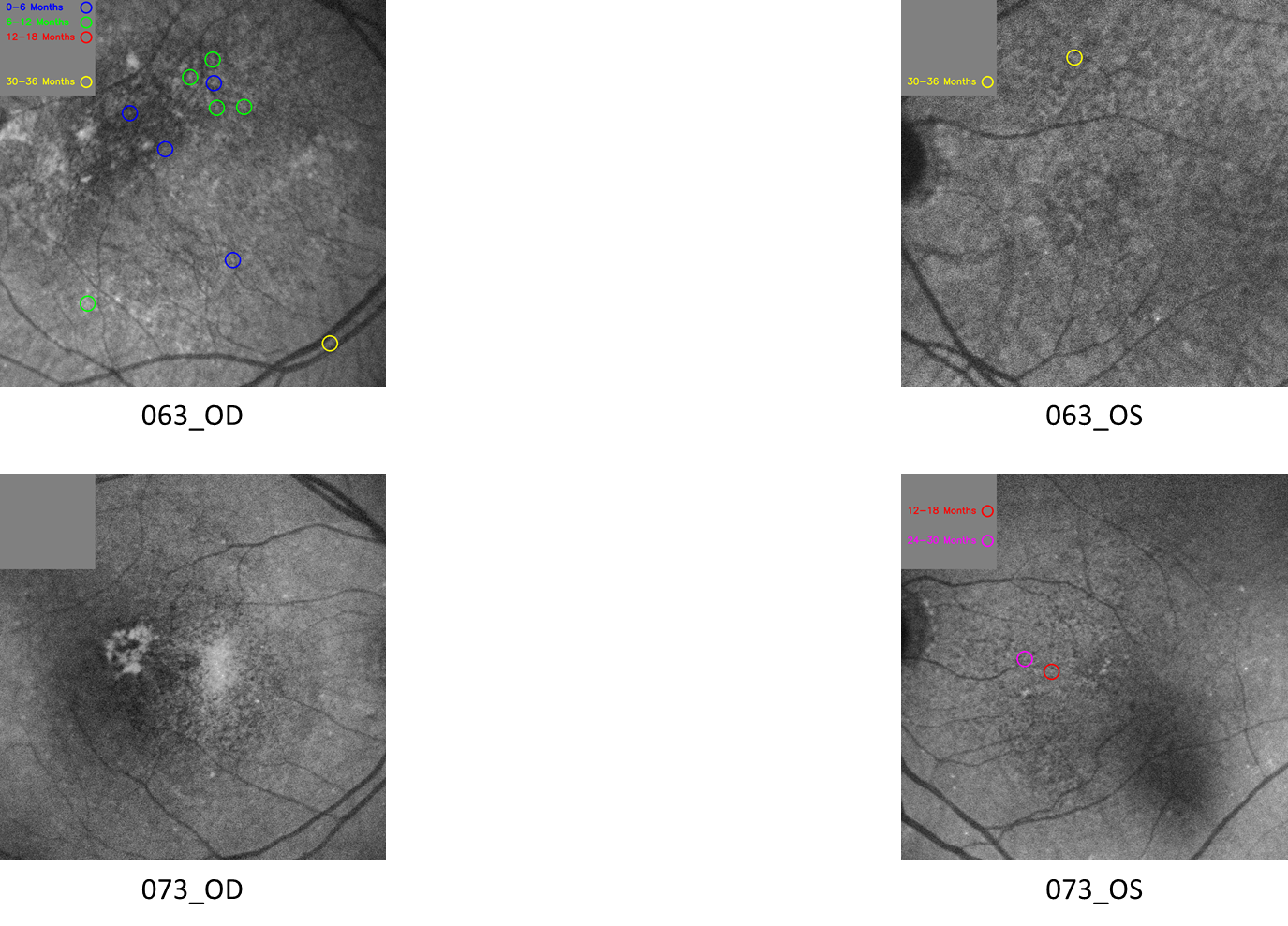


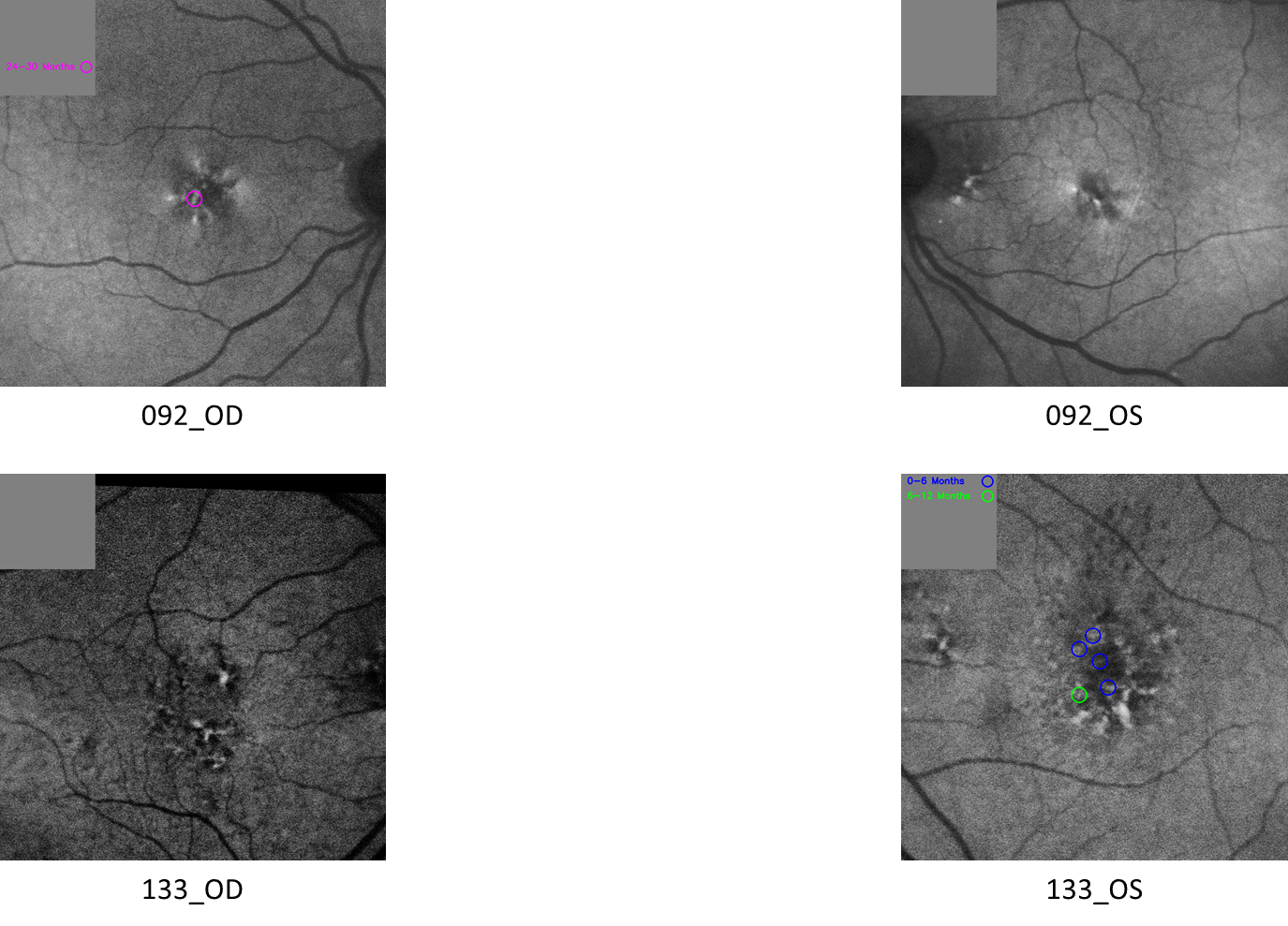

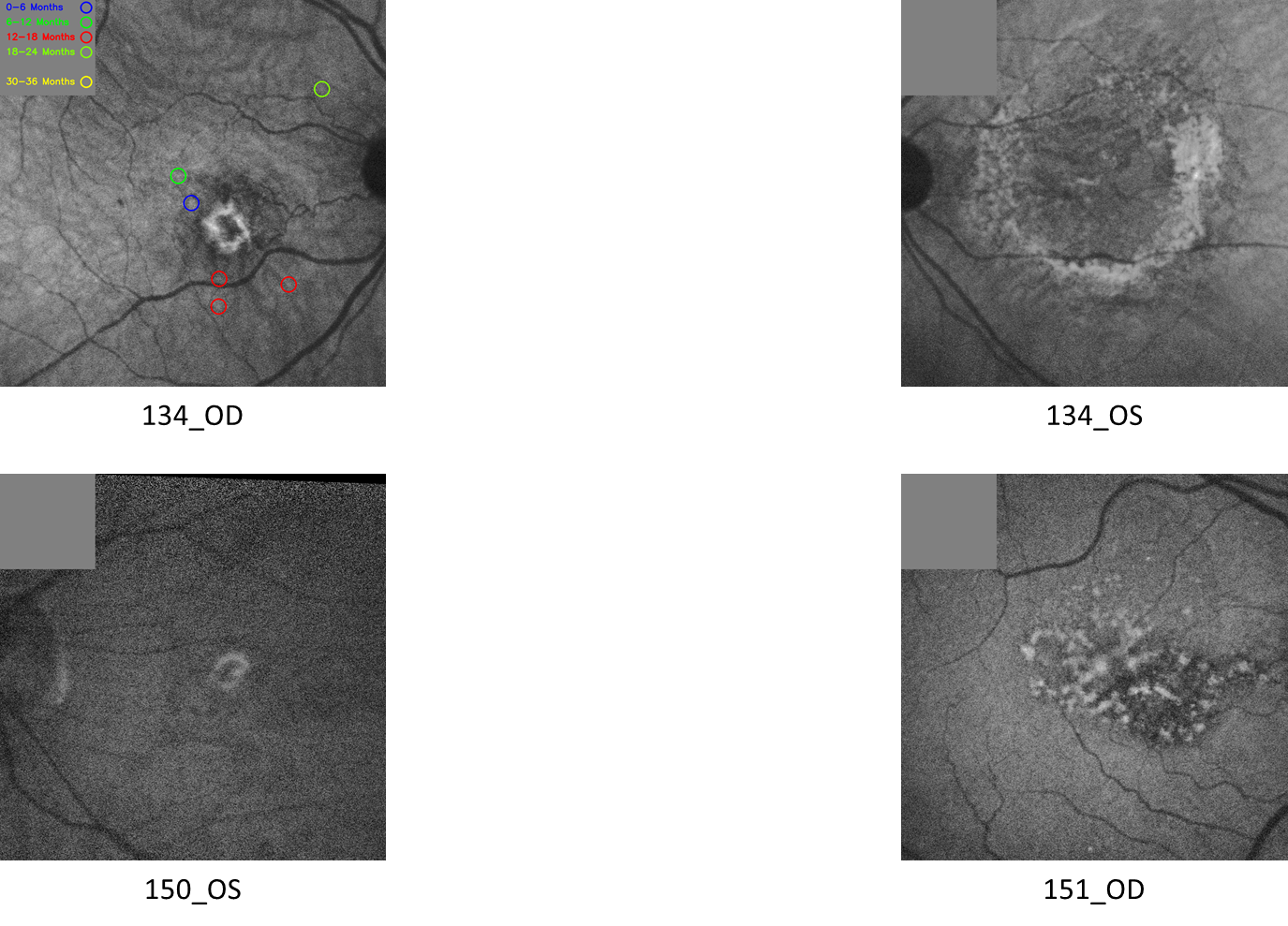


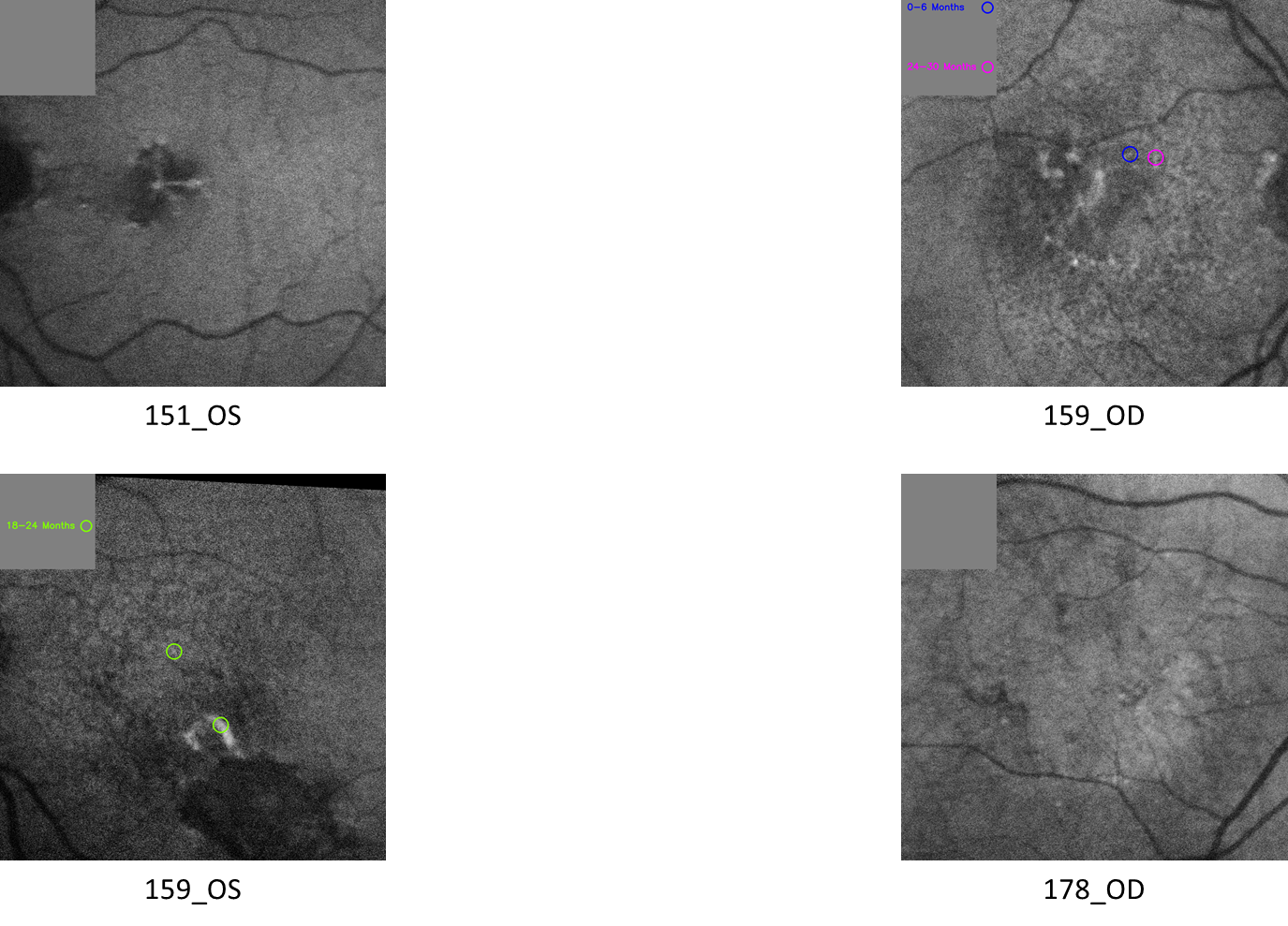


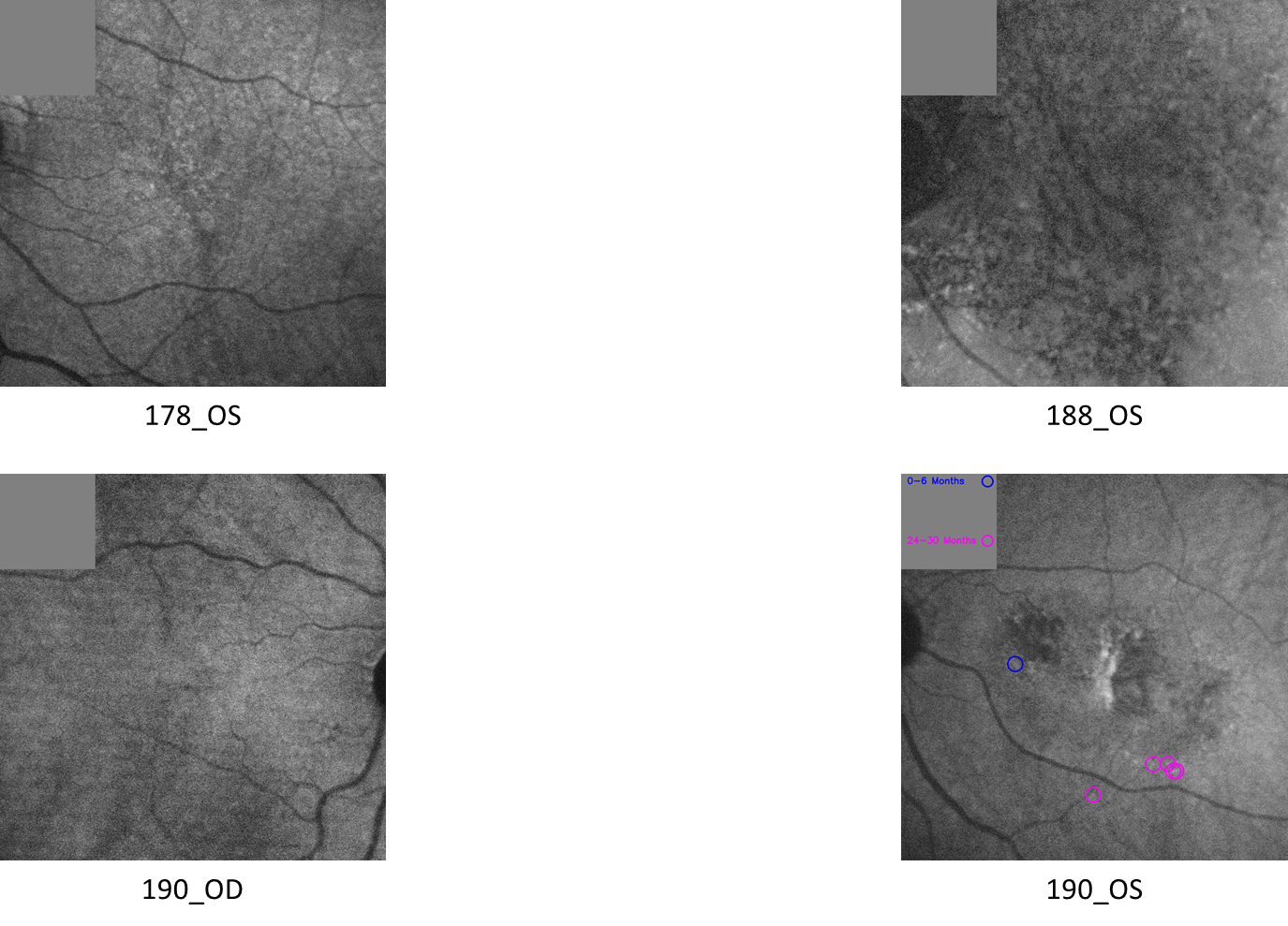


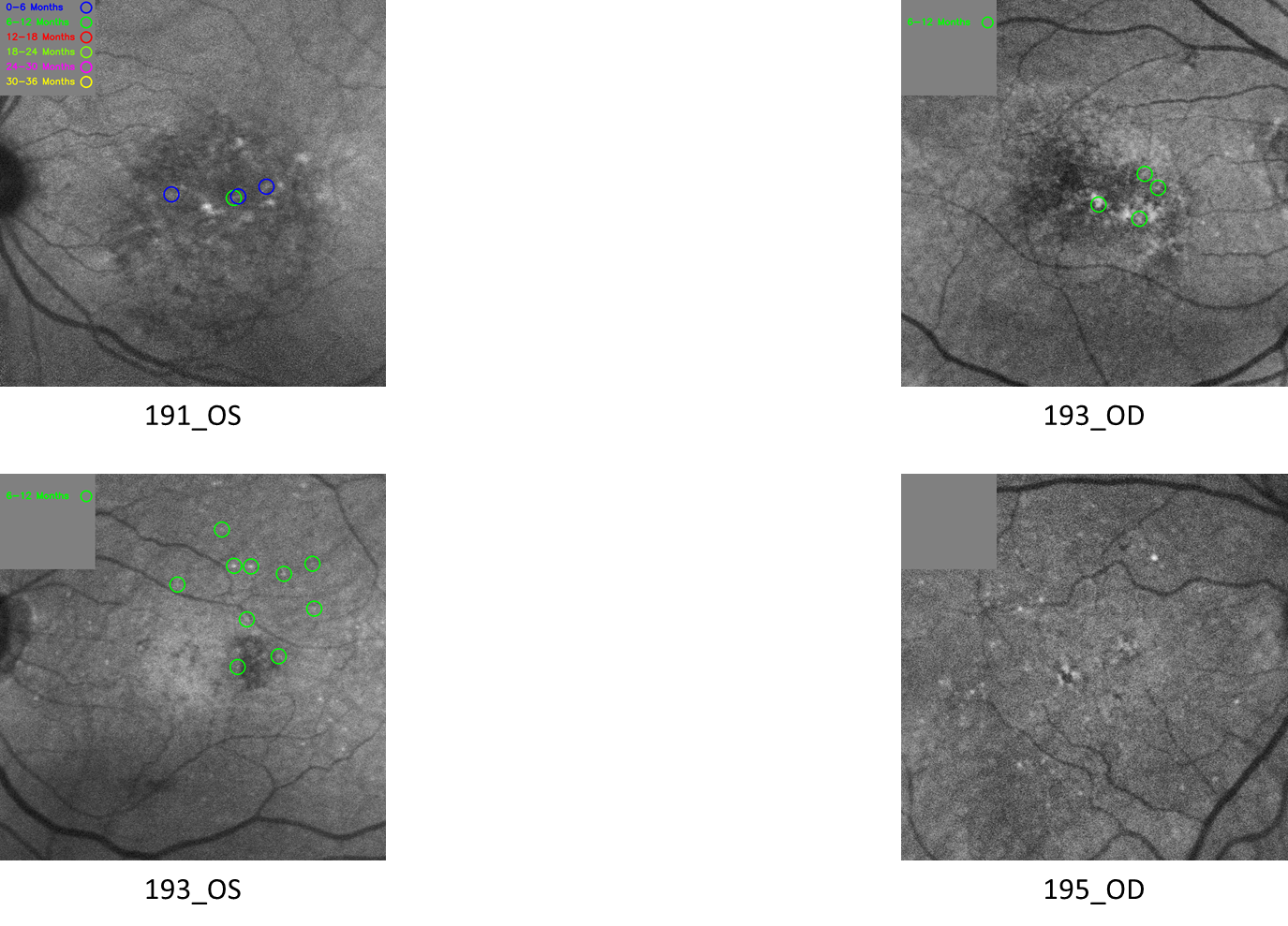


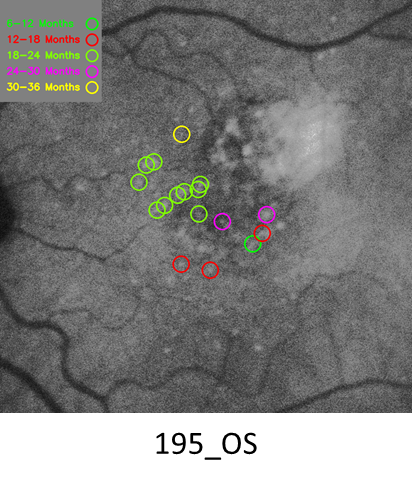


| Time (months) | | | | | | | |
| --- | --- | --- | --- | --- | --- | --- | --- |
|  | **Predictive Value**  **%** | **6** | **12** | **18** | **24** | **30** | **36** |
| DARC CNN | **PPV** | 71 | 70 | 82 | 77 | 86 | 80 |
|  | **Specificity** | 90 | 86 | 89 | 82 | 86 | 79 |
|  | **Sensitivity** | 83 | 88 | 82 | 83 | 80 | 80 |
|  | **NPV** | 95 | 95 | 89 | 88 | 80 | 79 |
| Yim 2020 | **PPV** | 7 | 16 |  | 33 |  |  |
|  | **Specificity** | 55 | 54 |  | 52 |  |  |
|  | **Sensitivity** | 80 | 80 |  | 80 |  |  |
|  | **NPV** | 98 | 10 |  | 90 |  |  |
| Bhuiyan 2020 | **Precision** |  | 25 |  | 24 |  |  |
|  | **Specificity** |  | 67 |  | 67 |  |  |
|  | **Sensitivity** |  | 74 |  | 71 |  |  |
|  | **Accuracy** |  | 68 |  | 67 |  |  |
| Banerjee 2019 | **Specificity** | 73 | 52 |  | 97 |  |  |
|  | **Sensitivity** | 80 | 80 |  | 80 |  |  |
|  | **AUC** | 83 | 77 |  | 89 |  |  |
| Russakoff 2019 | **Specificity** |  |  |  | 76 |  |  |
|  | **Sensitivity** |  |  |  | 80 |  |  |
|  | **AUC** |  |  |  | 79 |  |  |
| Schmidt-Erfurth 2018 | **Specificity** |  |  |  | 46 |  |  |
|  | **Sensitivity** |  |  |  | 80 |  |  |
|  | **AUC** |  |  |  | 68 |  |  |

## Supplementary Table 7: Prediction Values of CNV by AI Systems^10^ ^11^ ^9^ ^12^ ^13^

## References

1. Fasler K, Fu DJ, Moraes G, et al. Moorfields AMD database report 2: Fellow eye involvement with neovascular age-related macular degeneration. *Br J Ophthalmol*. 2020;104(5):684-690. doi:10.1136/bjophthalmol-2019-314446

2. Bek T, Klug SE. Incidence and risk factors for neovascular age-related macular degeneration in the fellow eye. *Graefe’s Arch Clin Exp Ophthalmol*. 2018:2061-2068. doi:10.1007/s00417-018-4100-z

3. Zarranz-Ventura J, Liew G, Johnston RL, et al. The neovascular age-related macular degeneration database: Report 2: Incidence, management, and visual outcomes of second treated eyes. *Ophthalmology*. 2014;121(10):1966-1975. doi:10.1016/j.ophtha.2014.04.026

4. Lee J, Choi S, Lee CS, et al. Neovascularization in Fellow Eye of Unilateral Neovascular Age-related Macular Degeneration According to Different Drusen Types. *Am J Ophthalmol*. 2019;208(June 2016):103-110. doi:10.1016/j.ajo.2019.07.013

5. Maguire MG, Daniel E, Shah AR, et al. Incidence of choroidal neovascularization in the fellow eye in the Comparison of Age-related Macular Degeneration Treatments Trials. *Ophthalmology*. 2013;120(10):2035-2041. doi:10.1016/j.ophtha.2013.03.017

6. Babenko B, Balasubramanian S, Blumer KE, et al. Predicting Progression of Age-related Macular Degeneration from Fundus Images using Deep Learning. April 2019. http://arxiv.org/abs/1904.05478. Accessed July 24, 2020.

7. Chakravarthy U, Bailey CC, Scanlon PH, et al. Progression from Early/Intermediate to Advanced Forms of Age-Related Macular Degeneration in a Large UK Cohort: Rates and Risk Factors. In: *Ophthalmology Retina*. Vol 4. Elsevier Inc; 2020:662-672. doi:10.1016/j.oret.2020.01.012

8. Marques JP, Costa M, Melo P, et al. Ocular Risk Factors for Exudative AMD: A Novel Semiautomated Grading System. *ISRN Ophthalmol*. 2013;2013:1-8. doi:10.1155/2013/464218

9. Banerjee I, de Sisternes L, Hallak J, et al. A Deep-learning Approach for Prognosis of Age-Related Macular Degeneration Disease using SD-OCT Imaging Biomarkers. February 2019. http://arxiv.org/abs/1902.10700. Accessed July 24, 2020.

10. Yim J, Chopra R, Spitz T, et al. Predicting conversion to wet age-related macular degeneration using deep learning. *Nat Med*. 2020. doi:https://doi.org/10.1038/s41591-020-0867-7

11. Bhuiyan A, Wong TY, Ting DSW, Govindaiah A, Souied EH, Smith RT. Artificial intelligence to stratify severity of age-related macular degeneration (AMD) and predict risk of progression to late AMD. *Transl Vis Sci Technol*. 2020;9(2):1-12. doi:10.1167/TVST.9.2.25

12. Russakoff DB, Lamin A, Oakley JD, Dubis AM, Sivaprasad S. Deep learning for prediction of AMD progression: A pilot study. *Investig Ophthalmol Vis Sci*. 2019;60(2):712-722. doi:10.1167/iovs.18-25325

13. Schmidt-Erfurth U, Waldstein SM, Klimscha S, et al. Prediction of Individual Disease Conversion in Early AMD Using Artificial Intelligence. *Invest Ophthalmol Vis Sci*. 2018;59(8):3199-3208. doi:10.1167/iovs.18-24106

14. Ronneberger O, Fischer P, Brox T. U-Net: Convolutional Networks for Biomedical Image Segmentation. :1-8.

15. Bogunovi H, Venhuizen F, Klimscha S, et al. RETOUCH - The Retinal OCT Fluid Detection and Segmentation Benchmark and Challenge. 2019;XX(XX).
